# Genetic Dissection of Alkalinity Tolerance at the Seedling Stage in Rice (*Oryza sativa*) Using a High-Resolution Linkage Map

**DOI:** 10.3390/plants11233347

**Published:** 2022-12-02

**Authors:** Lovepreet Singh, Sapphire Coronejo, Rajat Pruthi, Sandeep Chapagain, Uttam Bhattarai, Prasanta K. Subudhi

**Affiliations:** School of Plant, Environmental and Soil Sciences, Louisiana State University Agricultural Center, Baton Rouge, LA 70803, USA

**Keywords:** abiotic stress, candidate genes, genotyping-by-sequencing, Na^+^/K^+^ ratio, *Oryza sativa*, quantitative trait loci, salinity, single nucleotide polymorphism

## Abstract

Although both salinity and alkalinity result from accumulation of soluble salts in soil, high pH and ionic imbalance make alkaline stress more harmful to plants. This study aimed to provide molecular insights into the alkalinity tolerance using a recombinant inbred line (RIL) population developed from a cross between Cocodrie and Dular with contrasting response to alkalinity stress. Forty-six additive QTLs for nine morpho-physiological traits were mapped on to a linkage map of 4679 SNPs under alkalinity stress at the seedling stage and seven major-effect QTLs were for alkalinity tolerance scoring, Na^+^ and K^+^ concentrations and Na^+^:K^+^ ratio. The candidate genes were identified based on the comparison of the impacts of variants of genes present in five QTL intervals using the whole genome sequences of both parents. Differential expression of no apical meristem protein, cysteine protease precursor, retrotransposon protein, *OsWAK28*, MYB transcription factor, protein kinase, ubiquitin-carboxyl protein, and NAD binding protein genes in parents indicated their role in response to alkali stress. Our study suggests that the genetic basis of tolerance to alkalinity stress is most likely different from that of salinity stress. Introgression and validation of the QTLs and genes can be useful for improving alkalinity tolerance in rice at the seedling stage and advancing understanding of the molecular genetic basis of alkalinity stress adaptation.

## 1. Introduction

Abiotic stresses are major challenges for sustainable crop production worldwide. The ongoing climate change is exacerbating the frequency and severity of abiotic stresses. Alkaline stress suppresses plant growth, development, and productivity due to exposure to osmotic stress, nutritional deficiency, and ionic imbalance [1,2,3]. High pH and high concentration of salt (Na_2_CO_3_ or NaHCO_3_) impedes plant performance by interfering with the water absorption capacity caused by ionic and osmotic stresses [4,5,6,7]. Furthermore, precipitation of iron and phosphorus due to high pH around the rhizosphere results in nutritional deficiency in plants [2,8]. Alkaline stress causes severe damage to the root cells due to increased injury to membranes, increased malondialdehyde content, and upregulation of cell death-associated genes [9,10].

Rice (*Oryza sativa* L.) is one of the most important cereal crops worldwide. However, most cultivated rice varieties are susceptible to saline and alkaline stresses. Both stresses affect all stages of plant development starting from germination to the reproductive stage [11]. The intensity of plants’ injury depends on several factors including timing of stress and growth stages [12]. Alkaline stress is damaging to rice plants at both the seedling and reproductive stages [1,10,11]. Previous studies showed that there is either no or weak correlation between seedling and reproductive stage salinity tolerance [13,14] and therefore it has been suggested to screen the lines for salinity tolerance at the seedling stage followed by evaluation at the reproductive stage preferably under field conditions [15].

In saline-alkaline soil, soluble salts mostly consist of cations such as Na^+^, Ca^2+^, Mg^2+^, and K^+^ and anions like CO_3_^2−^, HCO_3_^−^, Cl^−^, SO_4_^2−^, and NO_3_^−^. Plants are more susceptible to alkalinity due to high pH and the presence of CO_3_^2−^ and HCO_3_^−^ [16]. However, the role of CO_3_^2−^ and HCO_3_^−^ in the severity of alkalinity is rarely emphasized. Moreover, osmotic stress, ionic stress, and high pH under alkaline stress reduce the iron (Fe) solubility [17]. As Fe in the soil exists in oxidized form (Fe^3+^), it becomes highly insoluble under aerobic conditions, especially at high pH [18], and plants show wilting and Fe deficiency symptoms [19]. Alkaline stress tolerance is a complex and polygenic trait [9,20,21], which makes developing alkalinity tolerant crop varieties challenging. Therefore, understanding the molecular basis of alkalinity tolerance is essential to make genetic progress.

The molecular genetic basis of salinity tolerance in rice has been investigated extensively compared to alkalinity tolerance [22,23,24]. The impacts of salinity have been evaluated at the morphological, physiological, and biochemical levels and genes contributing toward tolerance such as *SKC1* [25] and *DST* [26] have been identified. However, progress in understanding the molecular genetic basis of alkalinity tolerance is lagging behind. As both alkaline and saline stresses involve a high concentration of Na^+^ salt around the rhizosphere and in shoots, it has been assumed that there might be overlapping tolerance mechanisms for both stresses. Several studies showed that alkaline stress is more detrimental to crops compared with saline stress due to high pH and nutrient deficiency in addition to salt stress caused by Na_2_CO_3_ [9,27,28]. Apart from tolerance to ionic imbalance and osmotic stress, plants have evolved mechanisms for tolerating high pH and nutrient deficiency [29,30]. Various mechanisms such as ion exclusion at root level and its restricted transport to shoots and increased K^+^ uptake under stress environments have been reported to confer salinity tolerance in rice [31]. Few studies revealed that alkalinity tolerant cultivars possess genes for efficient acquisition of Fe and K under high pH [2,5,32]. Research efforts are needed to elucidate the mechanisms of the Na:K homeostasis, and Fe acquisition to enhance alkalinity tolerance in rice at the seedling stage.

The rapid development of genomics and molecular biology tools has enabled the dissection of the genetic basis of stress tolerance by identifying the major QTLs and genes to develop stress-tolerant rice cultivars using marker-assisted selection (MAS). A major alkaline tolerant QTL, *qARL11,* and two candidate genes from the F-box gene family, were identified using QTL mapping and a genome-wide association study [33]. Another major QTL *qSNC3* for alkaline stress tolerance explained 21% of phenotypic variation [30]. Few other studies reported QTLs at the germination and seedling stage of rice under alkaline stress [34,35,36]. Rice plants overexpressing the LSD1-like-type zinc finger protein OsLOL5 and nucleus-encoded thylakoid protein OsY3IP1 improved alkalinity tolerance [37,38]. Similarly, *Osppa6* was shown to play an important regulatory role in conferring alkalinity tolerance in rice [39]. However, there is no progress in the introgression of these QTLs/genes for rice improvement. In this study, we evaluated a recombinant inbred line (RIL) population and identified genomic regions for various morpho-physiological traits associated with alkaline tolerance in rice at the seedling stage. Since the next-generation sequencing has been used for genetic dissection of abiotic stress tolerance [22,40], we integrated results from both QTL mapping and whole genome sequencing (WGS) to identify candidate genes associated with alkaline tolerance in rice.

## 2. Results

### 2.1. Phenotypic Characterization under Alkaline Stress

**Figure 1:**
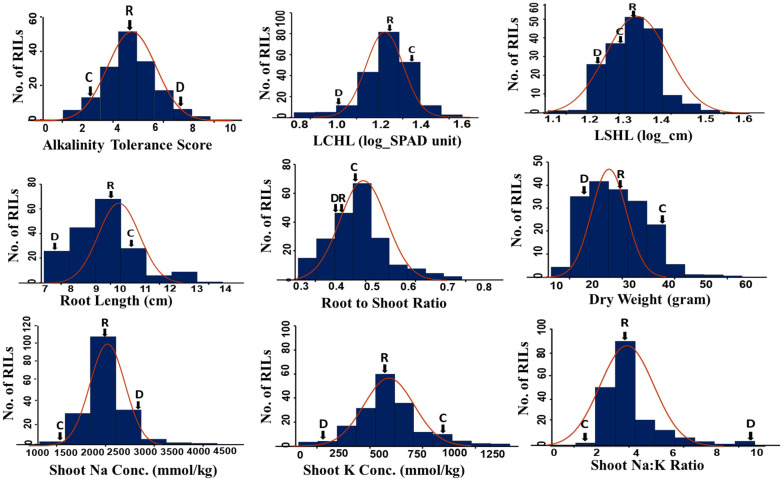
Frequency distribution of various morphological and physiological traits of Cocodrie × Dular RILs for alkalinity tolerance at the seedling stage with arrowheads indicating the trait means of Cocodrie (C), Dular (D), and RIL population (R). LCHL-log chlorophyll content; LSHL-log shoot length.

**Figure 2:**
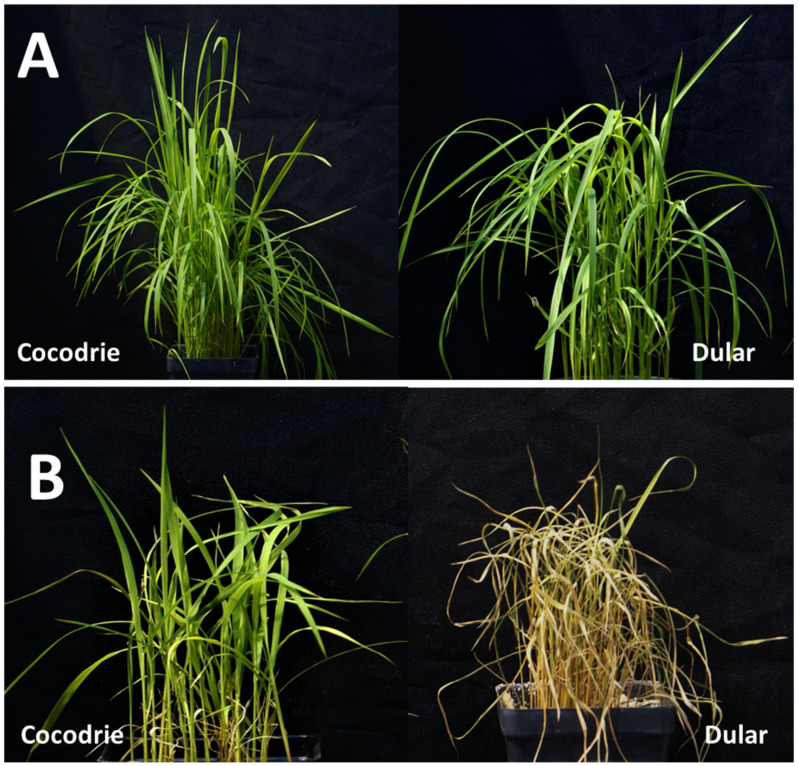
Performance of Cocodrie and Dular under control (**A**) and alkaline stress environments (**B**).

**Figure 3:**
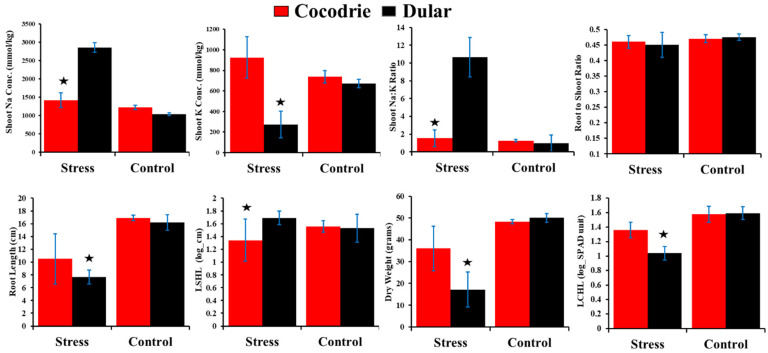
Comparison of Cocodrie and Dular under stress and control environment for alkalinity tolerance traits. Asterisks indicate significant difference between the means of Cocodrie and Dular under control and alkaline stress environment at 0.05 level of probability.

After three weeks of exposure to alkaline stress, the RILs and parents showed a varying level of tolerance based on observation of several traits such as alkalinity tolerance score (AKT), chlorophyll content (CHL), shoot length (SHL), root length (RTL), root to shoot ratio (RSR), shoot dry weight (DW), shoot Na^+^ concentration (SNC), shoot K^+^ concentrations (SKC), and Na^+^/K^+^ ratio (SNK) (Table 1). Except RSR, Cocodrie and Dular showed a significantly contrasting response for all other traits (Table 1 and Figure 1). AKT scores of Cocodrie and Dular were 3.7 and 7.7, respectively and both cultivars were scored 1 under control condition (Table S1 and Figure 2). All traits except CHL and SHL were normally distributed according to Shapiro–Wilk’s test and showed transgressive segregants on both sides of the distribution. The log transformed CHL (LCHL) and log transformed SHL (LSHL) data under alkaline stress were used for QTL mapping. On the contrary, all traits under the control condition were normally distributed (Figure S1). Cocodrie means were lower for AKT, SNC, and SNK but higher for SKC, LCHL, LSHL, RTL, DW, and RSR compared to Dular. Analysis of variance revealed significant differences among RILs for all traits except SNK (Table 1). The RIL mean values for all the traits were in between the mean values of Cocodrie and Dular except LSHL (Figure 2). The traits AKT, DW, RTL, RSR, SNC, and SKC showed moderate to high heritability, whereas SNK, LCHL, and LSHL exhibited low heritability. Heritability was significant and higher for all traits under control compared with the stress environment (Table S1). There was no difference for any morphological and physiological traits between Cocodrie and Dular under control condition (Figure 3).

### 2.2. Correlations among Traits in the RIL Population

The correlations among all the traits were presented in Table 2. There was a significant negative correlation between AKT and alkaline stress responsive traits such as RTL, DW, RSR, LSHL, LCHL, SKC, and SNK. However, it showed a significant positive correlation with SNC. SNC was only significantly negatively correlated with RTL and RSR. SKC had a significant negative correlation with AKT and a significant positive correlation with SNK, LCHL, RTL, and DW. RTL was significantly and positively correlated to SKC and RSR whereas it was negatively correlated with SNC and AKT. LSHL showed a significant positive correlation to RTL and DW, but a significant negative correlation with AKT and RSR.

### 2.3. Linkage Map and QTL Mapping

A total of 4679 SNP markers, generated by genotyping by sequencing, were used for linkage map construction. The linkage map covered 367 Mb of rice genome with a total genetic length of 1584 cM (Table 3). The average chromosome length was 132 cM with 3 SNPs per cM. The average number of gaps with more than 5 cM was 3.2 per chromosome.

#### 2.3.1. QTLs under Alkalinity Stress

**Figure 4:**
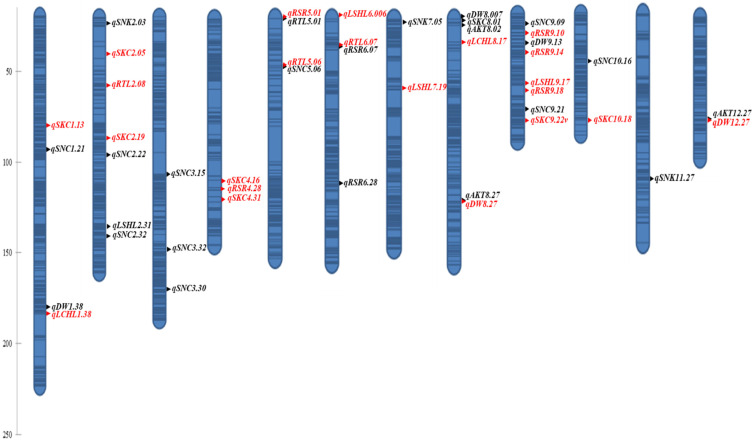
Positions of the QTLs on the linkage map for alkalinity tolerance traits in the Cocodrie × Dular RIL population. Red and blue fonts represent Cocodrie and Dular alleles for the increased means, respectively. Dark regions on the genetic map are the marker saturated regions and light regions represent gaps between the markers.

Forty-six QTLs were identified for various morphological and physiological traits under alkaline stress using inclusive composite interval mapping (ICIM) (Table 4 and Figure 4). Three QTLs were detected for AKT by ICIM and only *qAKT8.27* was a major effect QTL with a contribution of 11% toward phenotypic variation (PV). The Dular allele increased the AKT value for all the QTLs. Two QTLs were identified for LCHL with desirable alleles from Cocodrie in both cases. The *qLCHL1.38* was a major effect QTL with 27% of PV, while a minor effect QTL *qLCHL8.17* explained 6% of PV. A total of 4 QTLs were detected for LSHL. The large-effect QTL (*qLSHL2.31*) had Dular allele responsible for increased mean. All other QTLs were minor effect QTLs and the desirable allele for these QTLs was contributed by Cocodrie. For root length, four QTLs were identified. All QTLs except *qRTL5.01* were minor effect QTLs and had desirable alleles from Cocodrie, while *qRTL5.01* had desirable allele from Dular with a total PV of 10%.

There were three QTLs for RSR on chromosome 9, while two QTLs on chromosome 6 and one each on chromosome 5 and 4 under alkaline stress. Both *qRSR5.01* and *qRSR6.28* were large effect QTLs with a PV of 14% and 13%, respectively. The allele for increasing the mean at *qRSR5.01* was contributed by Cocodrie, while it was Dular allele for *qRSR6.28*. All other QTLs for RSR were small effect QTLs. Five QTLs were detected for shoot dry weight. All QTLs except *qDW12.27* were small effect QTLs. In case of three QTLs, Dular allele was responsible for the increased mean, while Cocodrie allele was responsible for the remaining two QTLs.

Na^+^ and K^+^ are the key factors determining alkalinity tolerance. For SNC, three large-effect QTLs and seven minor-effect QTLs were identified. All QTLs had alleles for increased mean contributed by Dular. The three major effect QTLs, *qSNC3.15*, *qSNC3.32*, and *qSNC10.16*, had a PV range of 10–13%. A total of seven QTLs were identified for SKC. Two major effect QTLs, *qSKC9.22* and *qSKC10.18*, explained 10% and 15% of PV, respectively. All other QTLs were small effect QTLs with two each detected on chromosomes 2 and 4 and one on chromosome 1. The alleles for increased mean in case of all QTLs were contributed by Cocodrie. For SNK, there were four QTLs and only *qSNK8.01* explained 10% of phenotypic variation whereas the remaining were minor effect QTLs with a PV value ranging from 4–9%. The desirable alleles for all QTLs were contributed by Dular. 

#### 2.3.2. QTLs under Control Environment

Twenty-six QTLs were identified for various morpho-physiological traits under the control environment by ICIM (Table S2). There was no QTL detected for AKT. Two and six QTLs were identified for CHL and SHL, respectively. The large-effect QTLs were *qCHL7.01*, *qSHL1.03,* and *qSHL9.18* with PV of more than 10%. Two QTLs each were detected for RTL and RSR. One QTL on chromosome 6, *qRSR6.13,* had a PV of 38%. For SNC and SKC, five QTLs for each trait were identified. All QTLs for SNC and SKC except *qSKC2.03* were small effect QTLs. The *qSKC2.03* explained 21% of PV. Four QTLs for SNK were detected and *qSNK7.05* was a large effect QTL. Incase of ten QTLs, Dular alleles were responsible for increased means, while Cocodrie alleles were contributed toward the increased means in the remaining QTLs.

### 2.4. Co-Localization of QTLs with Previous Salinity and Alkalinity Tolerance QTLs

Several QTLs from this study co-localized with earlier reported QTLs for salinity and alkalinity tolerance (Table 5). The interval of *qSKC1.13* QTL overlapped with relative root number QTL *qRRN1* detected under alkaline stress [41]. Both *qlogCHL1.28* and *qDW1.38* co-localized with the *qSHL1.38* identified in our previous study [36]. The QTLs, *qlogSHL2.31* and *qSNC2.32* located between 31–32 Mb position on chromosome 2 were present within the interval of earlier identified *qDLR2-1* [34]. Similarly, *qSNC2.22* and *qSKC2.19* were similar to the *qDLR2-2* and *qSNC3.15* was same as *qDLR3* [34]. The SNC QTLs, *qSNC3,* and *qSNC6* [30] under alkaline stress had an overlapping region with *qSNC3.32* and *qRSR6.28*, respectively, detected in this study. Furthermore, *qRKC3.32* and *qSNC4.16* [36] also co-localized with *qSNC.32* and *qSKC4.16* of this study. A shoot K^+^ conc. QTL, *qSKC4.31* co-localized with root length QTL *qARL4* under alkali stress [33]. Two QTLs on chromosome 5, *qRL5.06* and *qSNC5.06,* were located within the interval of *qDLRa5-1* under alkaline stress [35]. Both *qlogSHL6.006* and *qRSR6.28* were similar to the *qDLR6-1* detected under alkaline stress [34]. The *qDW8.12* and *qDW8.27* had an overlapping region with *qDSRs8-1* [35] and *qSHL8.27* [36], respectively. The QTLs, *qSNC10.16* and *qDW11.02,* identified in this study had an overlapped interval with *qRGE10* and *qRGE11*, respectively [41]. A major QTL *qSKC10.18* was also identified in the same genomic region in our previous study [36].

### 2.5. Candidate Genes Identification by Integrating Data from QTL Mapping and Whole Genome Sequencing

Using the flanking markers of QTLs, the genes present in the QTL intervals of the alkalinity tolerance traits were determined (Table S3). Five large effects QTLs (*qSNC3.32*, *qSNK8.01*, *qAKT8.27*, *qSKC9.22*, and *qSKC10.18*) were selected for detecting candidate genes based on polymorphic SNP and InDels present between parents. After removing SNPs and InDels in the intergenic, downstream, and upstream regions, all low, moderate, and high impact variants of genes present in the QTL intervals between Cocodrie and Dular were examined (Table S4). Only high-impact SNPs and InDels were considered and a total of 63 candidate genes were identified (Table S5). After removing the hypothetical genes and expressed proteins, the number of candidate genes was narrowed down to thirty (Table 6). There were twelve candidate genes within the interval of *qSNC3.32* based on the frameshift mutation, stop lost, stop gained, and splice donor variants differentiating Cocodrie and Dular. These genes were retrotransposon protein (LOC_Os03g56560 and LOC_Os03g57380), *ad-003* (LOC_Os03g56830), calreticulin family protein (LOC_Os03g59264), no apical meristem protein (LOC_Os03g59730), cys-rich domain-containing protein (LOC_Os03g61430), small heat shock protein (LOC_Os03g61940), hydrolase (LOC_Os03g62070), WD40-like Beta Propeller Repeat family protein (LOC_Os03g62370), MYB family transcription factor (LOC_Os03g62379), pentatricopeptide (LOC_Os03g62400) and OsWAK28-OsWAK receptor-like protein kinase (LOC_Os03g62430). In the case of *qSNK8.01*, seven genes were identified based on several stops gain, splice acceptor and donor, and frameshift variants and these were Cytochrome P450 (LOC_Os08g01470), NBS-LRR disease resistance protein (LOC_Os08g01580), *Rf1*, mitochondrial precursor (LOC_Os08g01640), dehydrogenase (LOC_Os08g01760), matrix attachment region binding protein (LOC_Os08g01810), CR4L subfamily gene (LOC_Os03g01830), and protein kinase family protein (LOC_Os08g02050). 

In the *qSKC9.22* interval, there were two candidate genes, vignain precursor (LOC_Os09g39090) and cysteine proteinase EP-B 1 precursor (LOC_Os09g39100), carrying only frameshift mutations. A transposon protein gene (LOC_Os08g44690) was detected within the interval of *qAKT8.27* based on the presence of frameshift and stop gain variants. Similarly, the presence of frameshift, stop gain, and splice acceptor variants between Cocodrie and Dular were detected within the interval of *qSKC10.18*. The candidate genes present in the *qSKC10.18* interval were phosphate translocator-related gene (LOC_Os10g34490), retrotransposon protein (LOC_Os10g34650), RIPER8 (LOC_Os10g34896), ubiquitin family protein (LOC_Os10g34960), ubiquitin-carboxyl extension (LOC_Os10g34990), semialdehyde dehydrogenase, NAD binding domain-containing protein (LOC_Os10g35170), Rf1, mitochondrial precursor (LOC_Os10g35640), and pentatricopeptide repeat domain-containing protein (LOC_Os10g35630).

### 2.6. Validation of Expression of Alkalinity Tolerance Related Genes 

**Figure 5:**
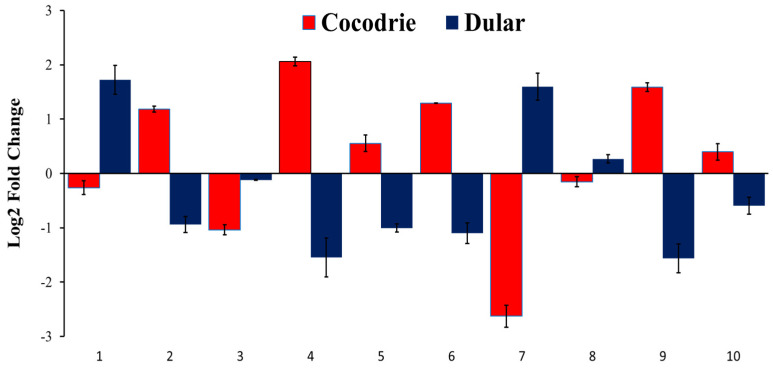
The expression level of selected genes in Cocodrie and Dular 6 h after exposure to alkalinity stress. *EF1α* was used as the reference gene. Log2 fold change was calculated for gene expression analysis under alkaline stress compared with control. 1–10 represents the genes used for expression analysis. 1-LOC_Os03g59730; 2-LOC_Os03g62430; 3-LOC_Os03g62370; 4-LOC_Os03g62379; 5-LOC_Os08g02050; 6-LOC_Os08g44690; 7-LOC_Os09g39100; 8-LOC_Os10g34650; 9-LOC_Os10g34490; 10-LOC_Os10g35170.

Ten genes were selected from the high-impact variants to determine their expression pattern under alkalinity stress compared to control (no stress) in rice by real-time reverse transcription PCR (qRT-PCR) (Table S6). The expression of LOC_Os03g59730 (no apical meristem protein), LOC_Os03g62370 (WD40-like beta propeller repeat family protein), LOC_Os09g39100 (cysteine protease EP-B 1 precursor), and LOC_Os10g34650 (retrotransposon protein) were downregulated in Cocodrie under alkaline stress whereas the expression level of these genes sharply increased in Dular 6 h after exposure to alkaline stress (Figure 5). In contrast, the expression of LOC_Os03g62430 (*OsWAK28*-OsWAK receptor-like protein kinase); LOC_Os03g62379 (MYB family transcription factor), LOC_Os08g02050 (protein kinase family protein), LOC_Os08g44690 (transposon protein), LOC_Os10g34490 (ubiquitin-carboxyl extension) and LOC_Os10g35170 (semialdehyde dehydrogenase, NAD binding domain-containing protein) were upregulated in Cocodrie compared to Dular under alkaline stress. 

## 3. Discussion

Saline and alkaline stress adversely affect the growth and development of rice plants resulting in reduced grain yield [35]. Compared with saline stress, alkalinity not only disrupts the ionic balance but also causes deficiency of essential nutrients and damages to cellular machinery due to high pH and the presence of carbonate cations [29,40]. Alkaline stress is more complicated than saline stress and deciphering the mechanisms of alkalinity tolerance at the seedling stage of rice is critical for improving rice production. Although several QTLs and candidate genes for alkalinity tolerance have been identified in rice [6,30,33,34,35,36,41,42], the molecular mechanism of alkalinity tolerance at the seedling stage in rice is not yet elucidated in greater detail. Thus, there is a need to unravel the molecular mechanisms and candidate genes associated with alkalinity tolerance in rice.

Alkaline stress reduced the growth and performance of all RILs. However, there was significant variation in the extent of damage among RILs for all traits except SNK (Table 1). Some RILs performed better than the parents under alkaline stress, suggesting the presence of transgressive segregants (Figure 2), which could be due to the complementary gene action of additive alleles dispersed in the parental lines [43]. The comparison of the performance of both parents under control and alkaline stress environment clearly showed that the impact of alkaline stress was more damaging to Dular than Cocodrie. Furthermore, there was a reduction in heritability estimates for all traits under alkaline stress compared to control suggesting the influence of alkaline stress on the expression of the traits as observed in earlier studies [44,45]. The traits with high to moderate heritability such as AKT and SNC could be exploited in breeding program for improving alkalinity tolerance in rice.

Correlation analysis is crucial to investigate the relationship among the physiological and morphological traits under stress conditions. Association of high AKT with high SNC in RILs suggesting inefficient exclusion of Na^+^ from roots and shoots was in agreement with earlier studies [46,47]. In susceptible lines, salt stress depolarizes the plasma membrane and reduces the K^+^ uptake by affecting the expression of K^+^ acquisition genes [48]. A negative and significant correlation between AKT and SKC in our study (Table 2) led to the same conclusion that tolerant RILs accumulated more potassium than susceptible lines. Tolerance was reflected by low AKT, low SNC, and high SKC. We observed a significant negative correlation between AKT and traits such as RTL, RSR, DW, LSHL, and LCHL. The association between root length and shoot length, chlorophyll, and dry weight were positive under the stress environment. An increase in root length under alkaline stress increased SHL, CHL, and DW. This suggests that a deep root system and high chlorophyll content under alkaline stress can improve alkalinity tolerance due to the ability to overcome osmotic stress and the nutrient deficiency in rice. Longer and deeper roots help to cope with high salt concentration and high pH near the surface and supply nutrients to the plants. A study showed that an approximately 20% increase in root surface under stress environment could mitigate stress by the instantaneous response of plants to uptake more nutrients and water [49]. Similarly, the transgenic rice overexpressing *OsMADS25* enhanced salt tolerance by developing deeper roots, while knock-down of *OsMADS25* increased salt sensitivity due to reduced root growth [50]. In this study, a significant negative association was found between SNC and SKC. Heritability for these physiological traits were low to medium in this study. In contrast to this study, a positive correlation between Na^+^ and K^+^ and high heritability of these traits were reported under saline stress [51]. However, results comparable to our study were reported in rice under alkaline stress [30]. Furthermore, Cocodrie was tolerant to alkali stress in contrast to its susceptibility to saline stress [52]. These findings suggested that alkaline tolerance mechanisms might be different from those for salinity tolerance to cope with the ionic imbalance and Fe deficiency under high pH.

We detected twenty-one QTLs for SNC, SKC, and SNK. Cocodrie alleles were responsible for increasing means at all SKC QTLs whereas Dular alleles increased means at all SNC and SNK QTLs. These findings signified the desirability of the Cocodrie allele to improve alkalinity tolerance. However, same tolerant parental allele contributed toward increased trait means at both Na^+^ and K^+^ QTLs under salt stress in an earlier study [53]. Therefore, it will be interesting to elucidate the underlying genes for Na^+^ and K^+^ accumulation in shoots under alkaline stress. Accumulation of K^+^ and maintenance of low Na^+^:K^+^ ratio and/or compartmentation of Na^+^ by Cocodrie could be the reason for alkalinity tolerance as reported in a previous study [5]. Most of the SNC QTLs of this study (*qSNC2.22*, *qSNC2.32*, *qSNC3.15*, *qSNC3.32*, *qSNC5.06*, and *qSNC10.16*) co-localized with the QTLs detected in previous alkaline stress studies (Table 5). The *qSNC3.32* co-localized with *qRKC3.32* identified in our earlier study [36]. Some SNC QTLs co-localized with the QTLs detected for morphological traits. These findings confirmed the association between physiological and morphological traits for stress tolerance. The *qSNC3.32* with a contribution of 13% of PV detected in this study co-localized with *qSNC3* [30] and two candidate genes (LOC_Os03g62500 and LOC_Os03g62620) in this QTL region showed differences in expression level between the parents under alkaline stress. It is possible that other genes within this genomic region might be responsible for alkalinity tolerance. This possibility was supported by our sequence analysis of *qSNC3.32* region which led to identification of more candidate genes downstream of the detected genes [30]. Furthermore, QTLs for Na^+^ concentration in the same region were detected under saline stress [54,55]. We further narrowed down the number of candidate genes to twelve within the *qSNC3.32* interval (Table 6). Receptor-like kinases play a significant role in plant signal transduction pathways. The upregulation of receptor kinase gene *OsWAK28* in Cocodrie compared to Dular (Figure 5) suggested the role of this gene in response to alkaline stress. In contrast to this study, the knockdown of *OsWAK112* improved rice salt tolerance which could be due to a different role in signal transduction process [56]. WD40 family protein (LOC_Os03g62370) was downregulated in Cocodrie compared to Dular (Figure 5). Contrary to our study, a rice WD40 protein gene *OsABT* was upregulated in rice under salt stress [57]. These results further corroborated our hypothesis of differences in tolerance mechanisms for alkalinity and salinity in rice. Transcription factors serve as a control switch for plant responses under stress environments [58]. The transcription factor genes, LOC_Os03g62379 and LOC_Os03g59730, were upregulated and downregulated, respectively in Cocodrie (Figure 5). In earlier transcriptomic studies, *OsMYB2P-1* [59] and *OsNAC52* [28] were differentially expressed between tolerant and sensitive cultivars under alkaline stress. These results suggest that MYB and NAC transcription factors play a crucial role in alkaline stress response and both LOC_Os03g62379 and LOC_Os03g59730 may be involved in regulating processes that affect tolerance to alkaline stress in rice, particularly at the seedling stage.

In case of SKC, *qSKC1.13*, *qSKC2.19*, *qSKC4.16*, *qSKC4.31* and *qSKC10.18,* co-localized with the QTLs identified under alkaline stress in earlier studies (Table 5). Both *qSKC9.22* and *qSKC10.18* were large effect QTLs. The *qSKC10.18* overlapped with an earlier reported QTL [36], while *qSKC9.22* was a novel QTL for alkalinity tolerance. In case of *qSKC9.22*, two potential candidate genes, LOC_Os09g39090 (vignain precursor) and LOC_Os09g39100 (cysteine protease EP-B 1 precursor) with high impact variants differentiated Cocodrie and Dular. Similarly, eight candidate genes were detected based on high-impact DNA polymorphisms between Cocodrie and Dular in case of *qSKC10.18* (Table 6). None of our SNK QTLs were same as earlier identified QTLs. The *qSNK8.01* was a large effect QTL and *qSNK2.03*, *qSNK7.05* and *qSNK11.27* were minor effect QTLs. Of particular interest is the *qSNK8.01* and sequence analysis narrowed the candidate genes number to seven within the QTL interval (Table 6). Most of the genes detected in *qSKC10.18* and *qSNK8.01* interval were not previously identified under alkaline stress. The LOC_Os09g39100 encoded a cysteine protease, related to cell apoptosis, which regulates cell death across the plant genome [60]. Damage to roots under alkaline stress in rice was associated with the upregulation of cell-death-related genes [10]. Cysteine protease was downregulated in Cocodrie and upregulated in Dular (Figure 5). Alkalinity tolerance of Cocodrie could be due to the suppression of cell death-related gene (LOC_Os09g39100) under high pH conditions. The expression of the ubiquitin-carboxyl gene (LOC_Os10g34990), semialdehyde dehydrogenase, NAD binding domain-containing protein (LOC_Os10g35170), and protein kinase family protein (LOC_Os08g02050) were upregulated in Cocodrie, while the expression of retrotransposon protein (LOC_Os10g34650) was reduced in Cocodrie under stress environment (Figure 5). Many studies demonstrated relationship between salinity tolerance and the protein ubiquitination process [61,62]. Similarly, semialdehyde dehydrogenase family genes, NAD binding protein, and retrotransposons have been reported to be responsive to abiotic stresses [63,64,65,66,67]. Since the role of these genes in alkaline stress response is unclear, these novel genes should be investigated in future for their potential in improving alkalinity tolerance in rice.

A total of twenty-five additive QTLs were detected for AKT, LCHL, LSHL, RTL, RSR, and DW. Except for LCHL and AKT, QTLs identified for each trait had varying contributions from Cocodrie and Dular. These results suggested contribution of both Cocodrie and Dular alleles to the expression of these traits. These observations reinforced the complex nature of the alkaline tolerance in rice and thus pyramiding of QTLs for different traits will be required to mitigate the impacts of alkalinity stress. The colocalization of *qAKT8.27* and *qDW8.27* in the same region as in the previous study [36] could be due to pleiotropic effect or tightly linked genes [68]. Similar results were observed in the case of *qDW1.38, qLCHL1.38*, *qLCHL1.38*, and *qDW1.38* which were congruent to *qSHL1.38* detected in our earlier study [36]. Selection of these QTLs could be helpful in breeding for alkalinity tolerance as selection for one trait will indirectly help select the other desirable traits. 

The alkalinity tolerance scores reflect the overall performance of plants under alkaline stress. Lines sensitive to alkaline stress showed a reduction in the shoot, root, dry weight, and chlorophyll content. The correlation analysis confirmed the negative association of AKT with these traits. In this study, Dular alleles were responsible for increasing the mean effects for all three QTLs (*qAKT8.02*, *qAKT8.27*, *qAKT12.27*). Therefore, a low AKT score and corresponding QTL allele from Cocodrie are desirable from the breeding perspective. None of these QTLs colocalized with the QTLs from earlier alkaline stress tolerance studies. This showed that these additive QTLs might be novel alkaline stress tolerance loci. In case of the major effect QTL *qAKT8.27,* sequence analysis delimited this region to only one transposon protein gene (LOC_Os08g44690). The transposons have been implicated in various plant stress responses [69,70,71,72]. The upregulation of LOC_Os08g44690 in our expression study suggested its role in phenotypic plasticity and stress adaptation. 

## 4. Conclusions

The present study demonstrated the power of integrating QTL mapping with next-generation sequencing to elucidate the complex mechanisms associated with alkalinity tolerance. In addition to identification of the genomic regions using a high-resolution genetic linkage map, the SNPs and InDels identified from the whole genome sequence analysis helped to select putative candidate genes associated with alkalinity tolerance and the differential expression of some of these genes demonstrated their role in response to alkaline stress. Despite the susceptibility of Cocodrie to salinity [52], it showed a high degree of tolerance to alkaline stress, suggesting different genetic mechanisms controlling tolerance to both stresses. The congruency of QTLs with earlier salt tolerance QTLs could be due to commonality in morpho-physiological response to salinity and alkalinity. However, analysis of the genome, transcriptome, and metabolome of genotypes with contrasting stress response at a global scale will provide insights into the mechanisms differentiating adaptation to salt and alkali stresses in the future. The QTLs and genes identified in this study can be useful for improving alkalinity tolerance in rice at the seedling stage and advancing understanding of the molecular genetic basis of alkalinity stress adaptation. 

## 5. Materials and Methods

### 5.1. Choice of Parents

Cocodrie and Dular were used as parents to develop a recombinant inbred line (RIL) population for this study. Cocodrie, a long grain rice cultivar released by the Louisiana State University Agricultural Center [73], is tolerant to alkaline stress [36]. Dular is an upland-adapted well-known donor for drought tolerance from India [74], but susceptible to alkaline stress. The individual plants of the F_2_ population developed from the cross between Cocodrie and Dular were advanced to F_8_ by the single seed descent method to develop a RIL population. 

### 5.2. Seedling Stage Screening for Alkalinity Tolerance

This experiment was set up in the LSU AgCenter greenhouse (Figure S2). A total of 189 RILs along with parents were screened for alkalinity tolerance at the seedling stage. A randomized complete block design (RCBD) for both control and stress experiments with three replications was used. Seeds were exposed to 50 °C for five days to break the dormancy. Fifteen seeds per replication were sown in the sand-filled “4 × 4” square pots. Plants were allowed to grow in the nutrient solution with pH 5.6 until two leaf stage. Nutrient solution was prepared by dissolving 1 g/L of Jack’s professional fertilizer (20-20-20) (J.R. Peters Inc., Allentown, PA, USA) and 300 mg/L ferrous sulfate. A protocol described by Singh et al. [36] was used for alkalinity tolerance screening. At the two-leaf stage, plants were exposed to alkaline stress of 0.20% and 0.40% sodium carbonate (Na_2_CO_3_) solution with pH 10 for the first two and third week, respectively. Plants in the control experiment were allowed to grow under normal conditions.

Only five uniform seedlings were used for morphological and physiological observations. Chlorophyll content (CHL) was measured using SPAD-50 chlorophyll meter (Spectrum Technologies, Inc., Aurora, IL, USA) 10 days after exposure to stress. When the sensitive parent Dular showed susceptibility symptoms related to alkaline stress, plants were scored visually for alkalinity tolerance (AKT) on a scale of 1 to 9 following Singh et al. [36]. Shoot length (SHL) was measured from the base of the tip of the seedling to the longest leaf. Root length (RTL) was recorded from the base of the culm to the tip of the root. Root-to-shoot ratio (RSR) was computed by dividing root length by shoot length. Five plants were oven-dried at 65 °C for five days and then shoot dry weight (DW) was obtained. For Na^+^ and K^+^, shoot samples were oven-dried at 65 °C for 10 days and 0.5 g of the ground sample from each line per replication was digested in 5 mL of HNO_3_ and 3 mL of H_2_O_2_ at 152–155 °C for 3 h [75]. The concentrations of shoot Na^+^ (SNC) and shoot K^+^ (SKC) from the digested samples were measured using a flame photometer (model PFP7, Bibby Scientific Ltd., Staffordshire, UK). Standard curves for Na^+^ and K^+^ were derived from the standard solutions of different dilutions. Then, the final concentration of Na^+^ and K^+^ were computed using standard curve readings. Shoot Na^+^:K^+^ ratio (SNK) was calculated by dividing the shoot Na^+^ concentration by shoot K^+^ concentration.

### 5.3. Statistical Analysis

R (version 2.2.1) and SAS software (version 9.4) were used for statistical analysis [76,77]. Shapiro–Wilk test was performed in R for each trait to assess the normality of the data and log transformation was used to transform the data for the traits that were not normally distributed. Descriptive statistics and histograms for each trait were obtained using the R. Aov function in R was used for computing the analysis of variance (ANOVA). To determine the relationship between morphological and physiological traits under alkaline stress, Pearson correlation coefficients were calculated in R. SAS was used to estimate broad sense heritability following Holland et al. [78].

### 5.4. Genotyping-by-Sequencing of the Mapping Population and SNP Identification

Leaf tissues were collected from 189 RILs and parents (Cocodrie and Dular) grown in the control environment. The genomic DNA was extracted using a modified CTAB method [79]. Extracted DNA was purified by the genomic DNA clean and concentrator kit (Zymo Research Corp. Irvine, CA, USA). The DNA concentration in each sample was estimated by Nanodrop ND-100 Spectrophotometer (Thermo Fisher Scientific, Wilmington, USA). The concentration of DNA was adjusted to 30–60 ng/µL for library construction. The protocol reported by Elshire et al. [80] was used for library preparation using the *ApeKI* restriction enzyme followed by single-end sequencing at the Genomic Diversity Facility of Cornell University. 

The TASSEL 3 GBS pipeline was used to process the raw sequence data [81]. Raw sequences without barcode were removed using the TASSEL plugin and good barcode reads were aligned to the Nipponbare reference genome using Bowtie 2 [82]. SNP calling and filtering were done using the TASSEL pipeline. The SNPs with high genotyping error, low coverage, and high heterozygosity were purged. Duplicate SNPs were merged. The remaining SNPs were manually filtered. SNPs markers with more than 10% missing alleles and SNPs with monomorphic alleles between both parents were discarded. The heterozygous SNPs were considered as missing data. 

### 5.5. Linkage Map Construction and QTL Mapping

IciMapping software v.4.1 was used for the construction of linkage map and QTL mapping [83]. Genotypic data were encoded as ‘2’, ‘0’, and ‘−1’ to represent Cocodrie, Dular, and missing alleles, respectively. The SNP markers were grouped based on their physical location on the chromosome. Recombination distance between the markers was calculated using the Kosambi mapping function [84] and was used for the ordering of markers within each chromosome. 

The mean value of three replications for each of the nine morphological and physiological traits was used for QTL analysis. Except for CHL and SHL, the data was normally distributed for the remaining seven traits and was used directly for QTL mapping. The data for CHL and SHL were log-transformed to improve the normality before QTL mapping and the traits were labeled as LCHL and LSHL, respectively. Inclusive composite interval mapping (ICIM) was used for detecting additive QTLs for alkalinity tolerance. To declare the significant additive QTLs, a scanning window size of every 1 cM with a 2.0 LOD score was used. The additive effect and phenotypic variation explained by each QTL were estimated. The positions of the right and left flanking markers were used to define the QTL intervals. The QTLs were named using trait name, followed by chromosome location and Mb position of the QTL. 

### 5.6. Whole Genome Resequencing of Parents and Detection of SNPs and InDels

The raw whole genome sequencing data of Dular (Accession number: PRJNA284427) was downloaded from National Center for Biotechnology Information (NCBI). Cocodrie was sequenced earlier in our lab at the School of Plant, Environment and Soil Sciences, LSU Agricultural Center and the whole genome sequencing data was submitted to NCBI (Accession number: PRJNA632686). The NGS QC toolkit (v2.3.3) was used for removing adapters and low-quality reads from the FASTQ files of Cocodrie and Dular. Reads with a Phred quality score of more than 30 were used for mapping using Burrows-Wheeler Alignment (BWA v0.7.17) [85,86]. SAMtools (v1.12) was then used to sort and convert the SAM files to BAM file [87]. Before variant calling, the BAM files were processed by Picard tools from the Genome Analysis Toolkit (GATK v4.0) [88]. The genomic variants present between two parents were identified using the Haplotype Caller tool in GATK [89]. VariantFiltration tool was used for stringent filtering and variants with quality depth (QD) below 2, strand odds ratio (SOR) above 3, fisher strand (FS) above 60, mapping quality (MQ) below 40, mapping quality tank sum (MQRankSum) below −12.5, and read position rank sum (ReadPosRankSum) below −8 for SNPs and QD below 2.0, FS above 200, and ReadPosRankSum below −20 for InDels, were removed. After the filtering, SnpEff v5.0e was used for annotation of the variants by selecting polymorphic alleles between Cocodrie and Dular, while sites with synonymous, upstream, downstream, intron, and intergenic variant effects were removed [90]. The physical locations of SNP markers flanking the major effect QTLs were used against the MSU Rice Genome Annotation release 7.0 to make an inventory of genes present within the QTL intervals.

### 5.7. Expression Analysis of Selected Genes by Real-Time Quantitative Reverse Transcription PCR (qRT-PCR) 

The seeds of both parents, Cocodrie and Dular, were incubated at 50 °C to break the dormancy and then germinated in Petri plates. After germination, twenty uniform seedlings per replication were transferred to a hydroponics setup using nutrient solution (1 g/L of 20-20-20 Jack’s professional fertilizer) at the LSU AgCenter greenhouse. The experiment was conducted with three replication for control and alkalinity stress. The seedlings were allowed to grow under normal conditions. The nutrient solutions were changed after every two days. At the two-leaf stage, plants in the control experiment were allowed to grow in normal conditions and seedlings in the stress experiment were exposed to alkaline stress (0.5% Na_2_CO_3_ with pH 10). Leaf samples were collected from both sets of experiments at 0 and 6 h of stress and immediately frozen in liquid N. Leaf samples were then stored at −80 °C until RNA extraction. Three biological replicates per treatment were used to extract total RNA using Trizol reagent. The quality and quantity of RNA were assessed in 1.2% agarose gel and a ND-1000 Spectrophotometer (Thermofisher Scientific, Waltham, MA, USA), respectively. The samples were then treated with PerfeCTa DNase 1 (Quantabio, Beverly, MA, USA) and iScipt™ first strand cDNA synthesis kit (Bio-Rad Laboratories, Hercules, CA, USA) was used to synthesize the first-strand cDNA following the manufacturer’s instructions. The genes for the expression analysis were selected from the whole genome sequence analysis. The primers for the qRT-PCR reaction were designed using PrimerQuest (Integrated DNA Technologies, Inc., Coralville, IA, USA) (Table S6). *EF1α* (LOC_Os03g08010) was used as an internal standard for expression normalization. The reactions were run in three technical replicates from the pooled cDNA samples of the biological replicates on an Applied Biosystems QuantStudio 3 Real-Time PCR System (Thermofisher Scientific, Waltham, MA, USA) using iTaq™ Universal SYBR Green Supermix (Bio-Rad Laboratories, Hercules, CA, USA) [91]. The expression level of genes was determined using 2^−∆∆CT^ method [92].

## Figures and Tables

**Table 1:** Means for various morphological and physiological traits in parents and Cocodrie × Dular RIL population under alkaline stress at the seedling stage.

Trait Name ^†^	Cocodrie Mean	Dular Mean ^§^	RIL Mean	Std. Dev.	RIL Range	RIL Pr > F ^$^	Heritability
AKT	3.7	7.7 *	5.2	1.8	1.0–9.0	<0.001 ***	0.81
LCHL	1.36	1.04 *	1.25	0.10	0.84–1.43	<0.001 ***	0.47
RTL (cm)	10.51	7.66 **	9.16	1.17	7.18–13.43	<0.001 ***	0.70
LSHL	1.30	1.20 ***	1.33	0.03	1.15–1.40	0.02 *	0.43
RSR (ratio)	0.48	0.45 ^ns^	0.47	0.07	0.31–0.71	<0.001 ***	0.61
DW (g)	36.0	17.1 **	26.9	7.52	11.3–50.9	<0.001 ***	0.77
SNC (mmol kg^−1^)	1418.3	2860.2 ***	2301.8	13.23	1095.5–4129.1	<0.001 ***	0.64
SKC (mmol kg^−1^)	925.4	274.1 ***	692.5	21.23	97.0–1884.7	0.01 **	0.65
SNK (ratio)	1.54	10.64 ***	3.64	16.84	1.10–21.16	0.52 ^ns^	0.46

^†^ AKT, alkalinity tolerance scoring; LCHL, log chlorophyll content; RTL, root length; LSHL, log shoot length; DW, shoot dry weight; RSR, root to shoot ratio; SNC, shoot Na^+^ concentration; SKC, shoot K^+^ concentration; SNK, shoot Na^+^:K^+^ ratio. ^§^ *t*-test between Cocodrie and Dular; ^$^ Genotypic difference between RILs based on ANOVA). *, **, *** Significant differences between the means Cocodrie and Dular at 0.05, 0.01, and <0.001 level of probability, respectively. ^ns^ Non-significant.

**Table 2:** Pearson correlation matrix of morphological and physiological traits measured in response to alkaline stress at seedling stage in Cocodrie × Dular RIL population.

Trait ^$^	AKT	LCHL	LSHL	RTL	DW	RSR	SNC	SKC	SNK
AKT	1.000								
LCHL	−0.037 ***	1.000							
LSHL	−0.038 **	0.052	1.000						
RTL	−0.026 **	0.027	0.100 *	1.000					
DW	−0.555 ***	0.298 ***	0.086 *	−0.072	1.000				
RSR	−0.009 **	−0.065	−0.658 ***	0.670 ***	0.006	1.000			
SNC	0.109 **	0.055	−0.05	−0.070 **	0.004	−0.015 *	1.000		
SKC	−0.101 **	0.131 ***	−0.004	0.134 **	0.109 **	−0.102	−0.017	1.000	
SNK	−0.006 **	0.602 ***	−0.051	−0.041	0.05	0.002 ***	0.075 **	0.127 **	1.000

^$^ AKT, alkalinity tolerance scoring; LCHL, log chlorophyll content; LSHL, log shoot length; RTL, root length; DW, shoot dry weight; RSR, root to shoot ratio; SNC, shoot Na^+^ concentration; SKC, shoot K^+^ concentration; SNK, shoot Na^+^:K^+^ ratio. * Significant at 0.05 level of probability; ** Significant at 0.01 level of probability; *** Significant at <0.001 level of probability.

**Table 3:** Distribution of SNP markers and genome coverage in the linkage map of Cocodrie × Dular RIL population.

Chr	No. of Markers	Chromosome Coverage (bp) ^$^	Genetic Length (cM) ^§^	No. of SNPs Per cM	Average Interval (cM)	No. of Gaps > 5 cM
1	646	43,172,608	215.8	2.99	0.83	5
2	511	35,280,580	150.8	3.39	0.91	3
3	600	36,110,022	194.7	3.08	0.87	3
4	360	33,875,971	132.9	2.71	1.11	5
5	214	29,764,180	130.5	1.64	1.32	2
6	309	31,099,571	130.6	2.36	1.05	4
7	453	29,302,669	124.4	3.64	0.82	4
8	292	28,135,784	133.3	2.19	1.09	3
9	355	22,692,796	87.8	4.04	0.78	1
10	298	21,675,087	83.1	3.58	0.87	1
11	308	28,904,361	116.9	2.63	1.27	5
12	333	27,448,210	83.6	3.98	0.89	2
Total	4679	367,461,839	1584.4	36.23	11.81	38
Mean	389.9	30,621,820	132.0	3.02	0.98	3.2

^$^ Physical length of chromosomes in base pairs (bp); ^§^ Length of chromosomes based on recombination events and measured in centimorgan (cM).

**Table 4:** Additive QTLs for traits related to alkaline tolerance at seedling stage in Cocodrie × Dular RIL population identified by ICIM method.

Trait ^$^	QTL ^§^	Chr	Position (cM)	Left_Marker	Right_Marker	Interval (bp)	LOD ^β^	PVE (%) ^ψ^	Additive Effect	Parental Allele with Increasing Effect
AKT	*qAKT8.02*	8	113	S8_2389044	S8_3753000	1,363,956	4.41	5.93	0.024	Dular
	*qAKT8.27*	8	131.5	S8_27687657	S8_28135748	448,091	7.17	10.71	0.300	Dular
	*qAKT12.27*	12	83.5	S12_27205921	S12_27488377	282,456	4.94	4.42	0.400	Dular
LCHL	*qLCHL8.17*	8	1	S8_1707207	S1_1929247	222,040	5.80	6.93	−0.026	Cocodrie
	*qLCHL1.38*	1	177	S1_38286772	S1_39460409	1,173,637	19.74	27.25	−0.008	Cocodrie
LSHL	*qLSHL2.31*	2	137	S2_31461037	S2_31512244	51,207	9.25	12.05	0.006	Dular
	*qLSHL6.006*	6	2.5	S6_690909	S6_1909168	1,218,259	4.85	6.13	−0.008	Cocodrie
	*qLSHL7.19*	7	69	S7_19188413	S7_19412780	224,367	8.39	9.26	−0.007	Cocodrie
	*qLSHL9.17*	9	59	S9_17841553	S9_18034390	192,837	2.11	3.44	−0.009	Cocodrie
RTL	*qRTL2.08*	2	54	S2_8268535	S2_8898321	629,786	2.53	5.96	−0.262	Cocodrie
	*qRTL5.01*	5	9.5	S5_1109689	S5_1278582	168,893	9.91	10.72	0.277	Dular
	*qRTL5.06*	5	40	S5_6448396	S5_6763151	314,755	8.33	8.91	−0.183	Cocodrie
	*qRTL6.07*	6	48	S6_7182868	S6_7971133	788,265	4.59	6.02	−0.262	Cocodrie
RSR	*qRSR4.28*	4	106.5	S4_28487153	S4_29482850	995,697	5.36	6.56	−0.013	Cocodrie
	*qRSR5.01*	5	8.5	S5_1065077	S5_1109689	44,612	10.50	14.82	−0.018	Cocodrie
	*qRSR6.07*	6	47.5	S6_7182868	S6_7971133	788,265	7.30	7.51	0.013	Dular
	*qRSR6.28*	6	117	S6_28968690	S6_30052494	1,083,804	11.66	13.08	0.014	Dular
	*qRSR9.10*	9	27	S9_10802084	S9_10898315	96,231	4.35	5.49	−0.013	Cocodrie
	*qRSR9.14*	9	41	S9_14050136	S9_14359383	309,247	2.62	4.98	−0.014	Cocodrie
	*qRSR9.18*	9	62.5	S9_18402360	S9_18643076	240,716	5.79	6.32	−0.016	Cocodrie
DW	*qDW1.38*	1	183	S1_38286772	S1_39460409	1,173,637	3.99	4.14	1.769	Dular
	*qDW8.007*	8	4	S8_799660	S8_1710677	911,017	6.61	7.74	1.444	Dular
	*qDW8.27*	8	131.5	S8_27687657	S8_28135748	448,091	8.07	9.77	−1.158	Cocodrie
	*qDW9.13*	9	39.5	S9_13908137	S9_13983427	75,290	6.99	8.31	1.588	Dular
	*qDW12.27*	12	82.5	S12_27205921	S12_27488377	282,456	7.58	10.61	−1.460	Cocodrie
SNC	*qSNC1.21*	1	94.5	S1_21692903	S1_21760129	67,226	5.94	8.25	5.933	Dular
	*qSNC2.22*	2	98.5	S2_22178643	S2_22249726	71,083	4.37	6.31	64.095	Dular
	*qSNC2.32*	2	144.5	S2_32743829	S2_32915982	172,153	6.38	9.42	65.049	Dular
	*qSNC3.15*	3	111	S3_15404332	S3_15513823	109,491	11.67	12.83	9.905	Dular
	*qSNC3.32*	3	155	S3_32028657	S3_35497210	3,468,553	9.96	13.28	5.252	Dular
	*qSNC3.30*	3	167	S3_30361166	S3_30382168	21,002	7.53	8.56	66.536	Dular
	*qSNC5.06*	5	44.5	S5_6833240	S5_7409167	575,927	3.16	5.08	60.171	Dular
	*qSNC9.09*	9	20	S9_9070610	S9_9106119	35,509	3.53	6.36	05.448	Dular
	*qSNC9.21*	9	76.5	S9_21162491	S9_21197575	35,084	5.81	7.07	76.133	Dular
	*qSNC10.16*	10	52.5	S10_16668990	S10_16691548	22,558	8.23	10.08	59.920	Dular
SKC	*qSKC1.13*	1	80	S1_13776034	S1_13961648	185,614	14.02	8.94	−220.509	Cocodrie
	*qSKC2.05*	2	38.5	S2_5560927	S2_5587375	26,448	9.08	6.50	−35.930	Cocodrie
	*qSKC2.19*	2	85	S2_19229724	S2_19269684	39,960	7.20	8.28	−27.125	Cocodrie
	*qSKC4.31*	4	117.5	S4_31786637	S4_32369172	582,535	6.52	7.34	−29.889	Cocodrie
	*qSKC4.16*	4	131.5	S4_16479675	S4_17381790	902,115	4.31	6.31	−28.608	Cocodrie
	*qSKC9.22*	9	86.5	S9_22415213	S9_22452432	37,219	8.65	10.37	−32.689	Cocodrie
	*qSKC10.18*	10	99	S10_18317578	S10_19335407	1,017,829	12.92	15.63	−11.789	Cocodrie
SNK	*qSNK2.03*	2	19.5	S2_3263428	S2_4041906	778,478	11.92	4.71	0.249	Dular
	*qSNK7.05*	7	34	S7_5858227	S7_6031361	173,134	6.23	7.91	0.195	Dular
	*qSNK8.01*	8	88.5	S8_261276	S8_799660	538,384	6.72	10.63	0.297	Dular
	*qSNK11.27*	11	116.5	S11_27210387	S11_28904361	1,693,974	8.37	9.21	0.205	Dular

^$^ AKT, alkalinity tolerance scoring; LCHL, log chlorophyll content; LSHL, log shoot length; RTL, root length; RSR, root to shoot ratio; SNC, shoot Na^+^ concentration; SKC, shoot K^+^ concentration; SNK, shoot Na^+^:K^+^ ratio. ^§^ *qAKT*, *qLCHL*, *qLSHL*, *qRTL*, *qRSR*, *qDW, qSNC*, *qSKC*, and *qSNK* are QTLs for alkalinity tolerance, log chlorophyll content, log shoot length, root length, root to shoot ratio, dry weight, shoot Na^+^ concentration, shoot K^+^ concentration, and shoot Na^+^:K^+^ ratio, respectively. The number before the decimal represents the chromosome number and the number after the decimal indicates the physical position of the QTLs in mega base pair. ^β^ LOD logarithm of odds. ^ψ^ PVE (%) percentage phenotypic variance explained by the QTL.

**Table 5:** Summary of additive QTLs co-localized with QTLs detected in previous alkalinity tolerance studies.

This Study			Previous Studies	
QTLs ^†^	Position	QTL	Flanking Markers and/or Position	References
*qDW8.12*	12,384,756–12,458,250	*qDSRs8-1*	RM22741 (9,957,711)-RM404 (15,438,081)	[35]
*qDW8.27*	27,687,657–28,135,748	*qSHL8.27*	27,384,352–27,875,737	[36]
*qDW11.02*	2,360,859–3,182,929	*qRGE11*	RM1812 (2,405,106)-RM5599 (3,824,353)	[41]
*qLCHL1.38* *qDW1.38*	38,286,772–39,460,409	*qSHL1.38*	38,286,772–38,611,845	[36]
*qLSHL6.006*	690,909–1,909,168	*qDLR6-1*	RM584 (441,616)-RM225 (3,416,533)	[34]
*qRSR6.28*	28,968,690–30,052,494	*qSNC6*	RM20517 (27,113,843)-RM412 (30,328,051)	[30]
*qLSHL2.31* *qSNC2.32*	31,461,037–31,512,244 32,743,829–32,915,982	*qDLR2-1*	RM5425 (28,267,534)-RM406 (35,236,078)	[34]
*qSNC3.15*	15,404,332–15,513,823	*qDLR3*	RM338 (13,221,482)-RM2453 (20,243,819)	[34]
*qSNC3.32*	32,028,657–35,497,210	*qRKC3.32* *qSNC3*	32,785,101–36,366,411 RM1221 (33,386,334)-RM130 (35,669,797)	[36] [30]
*qSNC2.22* *qSKC2.19*	22,178,643–22,249,726 19,229,724–19,269,684	*qDLR2-2*	RM29 (17,484,665)-RM221 (27,610,063)	[34]
*qRTL5.06* *qSNC5.06*	6,448,396–6,763,151 6,833,240–7,409,167	*qDLRa5-1*	RM243 (2,212,736)-RM413 (7,970,722)	[35]
*qSNC10.16*	16,668,990–16,691,548	*qRGE10*	RM467 (13,488,471)-RM271 (22,243,349)	[41]
*qSKC1.13*	13,776,034–13,961,648	*qRRN1*	RM1 (4,635,793)-RM195 (21,475,599)	[41]
*qSKC4.16*	16,479,675–17,381,790	*qSNC4.16*	16,612,171–16,880,788	[36]
*qSKC4.31*	31,786,637–32,369,172	*qARL4*	32,090,432–32,195,798	[33]
*qSNK8.01*	261,276–498,009	*qMT8.002* ^§^	261,276–799,660	[36]
*qSKC10.18*	18,317,578–19,335,407	*qSKC10.18*	18,053,155–19,335,416	[36]

^†^*qLSHL*, *qRTL*, *qRSR*, *qDW, qSNC, qSKC* are QTLs for log shoot length, root length, root to shoot ratio, dry weight, shoot Na^+^ concentration, and shoot K^+^ concentration, respectively. ^§^ *qMT8.002* represents multiple QTLs (*qAKT8.002*, *qSNC8.002*, *qRNC8.002*, *qSKC8.002*, *qRKC8.002*, *qSNK8.002*, *qRNK8.002)* mapped on to the same genomic region.

**Table 6:** List of polymorphic high impact SNPs and InDels in the genomic regions of selected additive QTLs.

QTL ^$^ and MSU Locus ID	Position ^ψ^	Cocodrie Allele	Dular Allele	SNPs/InDels Annotation ^§^	Molecular Function
*qSNC3.32* (LOC_Os03g56560)	32,220,387	G	A	SL	retrotransposon protein, putative, unclassified, expressed
	32,242,771	G	T	SG	
	32,243,087	T	TC	FS	
*qSNC3.32* (LOC_Os03g56830)	32,380,753	ACCAAGGTCTC	A	FS	*ad-003*, putative, expressed
*qSNC3.32* (LOC_Os03g57380)	32,727,633	G	A	SG	retrotransposon protein, putative, unclassified, expressed
	32,727,672	CA	C	FS	
	32,728,593	GA	G	FS	
*qSNC3.32* (LOC_Os03g59264)	33,744,655	GTT	G	FS	calreticulin family protein, expressed
	33,744,664	C	CA	FS	
	33,744,981	C	T	SD	
*qSNC3.32* (LOC_Os03g59730)	34,005,054	CT	C	FS	No apical meristem protein, putative, expressed
	34,005,056	GCT	G	FS	
*qSNC3.32* (LOC_Os03g61430)	34,857,333	G	GGT	FS	uncharacterized Cys-rich domain containing protein, putative, expressed
	34,857,738	GGT	G	FS	
*qSNC3.32* (LOC_Os03g61940)	35,113,812	C	CA	FS	small heat shock protein, chloroplast precursor, putative, expressed
	35,113,814	C	CCTA	SG	
	35,114,105	GC	G	FS	
*qSNC3.32* (LOC_Os03g62070)	35,167,884	T	TG	FS	hydrolase, putative, expressed
*qSNC3.32* (LOC_Os03g62370)	35,332,325	GC	G	FS	WD40-like Beta Propeller Repeat family protein, expressed
	35,332,328	GCC	G	FS	
*qSNC3.32* (LOC_Os03g62379)	35,337,103	A	G	SL	MYB family transcription factor, putative, expressed
*qSNC3.32* (LOC_Os03g62400)	35,348,023	A	T	SG	pentatricopeptide, putative, expressed
*qSNC3.32* (LOC_Os03g62430)	35,364,182	A	AT	FS	OsWAK28-OsWAK receptor-like protein kinase, expressed
	35,364,457	T	TA	SD	
*qSNK8.01* (LOC_Os08g01470)	293,465	G	T	SG	cytochrome P450, putative, expressed
	294,544	C	A	SA	
*qSNK8.01* (LOC_Os08g01580)	344,770	G	A	SG	NBS-LRR disease resistance protein, putative, expressed
*qSNK8.01* (LOC_Os08g01640)	374,325	A	T	FS	Rf1, mitochondrial precursor, putative, expressed
*qSNK8.01* (LOC_Os08g01760)	458,977	G	CCG	SD	dehydrogenase, putative, expressed
*qSNK8.01* (LOC_Os08g01810)	490,163	CCTAG	C	FS	matrix attachment region binding protein, putative, expressed
	490,545	TC	T	FS	
*qSNK8.01* (LOC_Os08g01830)	498,550	G	T	SG	TKL_IRAK_CR4L.6-The CR4L subfamily has homology with Crinkly4
*qSNK8.01* (LOC_Os08g02050)	665,685	C	A	SA	protein kinase family protein
*qAKT8.27* (LOC_Os08g44690)	28,085,435	T	C	SG	transposon protein, putative, Pong sub-class, expressed
	28,086,068	AAGAAGGAACAAACT	A	FS	
*qSKC9.22* (LOC_Os09g39090)	22,438,234	TCTTAACGATCC	TCTTAACGATCC	FS	vignain precursor, putative, expressed
	22,438,659	^α^32-bp	G	FS	
	22,438,815	ATCGCGTTGATCCCC	A	FS	
	22,439,053	GCCATTCAGTCT	G	FS	
	22,439,318	GACGGCGCGTAC	G	FS	
*qSKC9.22* (LOC_Os09g39100)	22,448,199	ACACGCTGCACCAC	A	FS	cysteine protease EP-B 1 precursor
*qSKC10.18* (LOC_Os10g34490)	18,402,218	A	G	FS	phosphate translocator-related, putative, expressed
	18,403,126	G	GCGCTCAC	SG	
*qSKC10.18* (LOC_Os10g34650)	18,463,513	G	T	SG	retrotransposon protein
*qSKC10.18* (LOC_Os10g34896)	18,628,571	C	G	SA	RIPER8-Ripening-related family protein precursor
	18,629,118	CGCCGAGGCGCA	C	FS	
*qSKC10.18* (LOC_Os10g34960)	18,658,668	^α^23-bp	C	FS	ubiquitin family protein, putative, expressed
*qSKC10.18* (LOC_Os10g34990)	18,666,630	TCTTC	T	FS	ubiquitin-carboxyl extension, putative, expressed
	18,666,647	A	ATGCT	FS	
*qSKC10.18* (LOC_Os10g35170)	18,772,532	C		SG	semialdehyde dehydrogenase, NAD binding domain containing protein, putative, expressed
*qSKC10.18* (LOC_Os10g35640)	19,057,187	T	TCC	FS	Rf1, mitochondrial precursor, putative, expressed
*qSKC10.18* (LOC_Os10g35630)	19,105,088	G	GAT	FS	pentatricopeptide repeat domain containing protein

^$^*qSNC*, *qSNK*, *qAKT*, *qSKC* are QTLs for shoot Na^+^ concentration, Alkalinity tolerance scoring and shoot Na^+^:K^+^ ratio, Shoot K^+^ concentration, respectively. The number before the decimal represents the chromosome number and the number after the decimal indicates the physical position of the QTLs in mega base pair. **^ψ^** Physical position based on IRGSP 1.0; **^§^** FS: Frame shift, SG: Stop gained, SL: Stop lost, SA: Splice acceptor, SD: Splice donor. ^α^32-bp (GGGTAGTAGGGAAGCTTGCCGCATTGGTCCAA); ^α^23-bp (CCCTTCTCCCCGCCGGTCACCAT).

## Data Availability

The data presented in this study are available in the article and Supplementary Material.

## Supplementary Materials

The following supporting information can be downloaded at: https://www.mdpi.com/article/10.3390/plants11233347/s1, Table S1. Mean values for various morpho-physiological traits at the seedling stage under control environment in Cocodrie × Dular RIL population; Table S2. Additive QTLs for seedling stage morpho-physiological traits under control environment identified by ICIM method in Cocodrie × Dular RIL population; Table S3. List of List of genes present in QTL intervals of alkalinity tolerance traits; Table S4. List of candidate genes with high impact variants; Table S5. Polymorphic low, moderate, and high impact variants between Cocodrie and Dular for five selected alkalinity tolerance QTLs; Table S6. List of primers used for gene expression by quantitative real-time reverse transcription PCR (qRT-PCR); Figure S1. Frequency distribution of various morphological and physiological traits of Cocodrie × Dular RILs under a control condition at the seedling stage with arrowheads indicating the trait means of Cocodrie (C), Dular (D), and RIL population (R). CHL—chlorophyll content; SHL—shoot length; RTL—root length; RSR—root to shoot ratio; DW—shoot dry weight; SKC—shoot K^+^ concentration; SNC—shoot Na^+^ concentration; SNK—shoot Na^+^:K^+^ ratio.; Figure S2. Alkalinity tolerance screening of RIL mapping population and parents in sand culture in the greenhouse experiment. A—Experimental setup for alkalinity stress screening at the seedling stage; B—one week old seedlings after germination; C—Performance of RILs under control experiment; D—Performance of RILs after 2 weeks of exposure to alkalinity stress.

## References

[1] Wei L.X., Lv B.S., Wang M.M., Ma H.Y., Yang H.Y., Liu X.L., Jiang C.J., Liang Z.W. (2015). Priming effect of abscisic acid on alkaline stress tolerance in rice (*Oryza sativa* L.) seedlings. Plant Physiol. Biochem..

[2] Li Q., Yang A., Zhang W.H. (2016). Efficient acquisition of iron confers greater tolerance to saline-alkaline stress in rice (*Oryza sativa* L.). J. Exp. Bot..

[3] Lu X., Min W., Shi Y., Tian L., Li P., Ma T., Zhang Y., Luo C. (2022). Exogenous melatonin alleviates alkaline stress by removing reactive oxygen species and promoting antioxidant defense in rice seedlings. Front. Plant Sci..

[4] Chen H., Zhang Q., Cai H., Xu F. (2017). Ethylene mediates alkaline-induced rice growth inhibition by negatively regulating plasma membrane H^+^-ATPase activity in roots. Front. Plant Sci..

[5] Chuamnakthong S., Nampei M., Ueda A. (2019). Characterization of Na^+^ exclusion mechanism in rice under saline-alkaline stress conditions. Plant Sci..

[6] Li N., Zheng H., Cui J., Wang J., Liu H., Sun J., Liu T., Zhao H., Lai Y., Zou D. (2019). Genome-wide association study and candidate gene analysis of alkalinity tolerance in *japonica* rice germplasm at the seedling stage. Rice.

[7] Neina D. (2019). The role of soil pH in plant nutrition and soil remediation. Appl. Environ. Soil Sci..

[8] Tian Z., Li J., Jia X., Yang F., Wang Z. (2016). Assimilation and translocation of dry matter and phosphorus in rice genotypes affected by salt-alkaline stress. Sustainability.

[9] Lv B.S., Li X.W., Ma H.Y., Sun Y., Wei L.X., Jiang C.J., Liang Z.W. (2013). Differences in growth and physiology of rice in response to different saline-alkaline stress factors. Agron. J..

[10] Zhang H., Liu X.L., Zhang R.X., Yuan H.Y., Wang M.M., Yang H.Y., Ma H.Y., Liu D., Jiang C.J., Liang Z.W. (2017). Root damage under alkaline stress is associated with reactive oxygen species accumulation in rice (*Oryza sativa* L.). Front. Plant Sci..

[11] Rao P.S., Mishra B., Gupta S.R., Rathore A. (2008). Reproductive stage tolerance to salinity and alkalinity stresses in rice genotypes. Plant Breed..

[12] Sangwongchai W., Krusong K., Thitisaksakul M. (2021). Salt tolerance at vegetative stage is partially associated with changes in grain quality and starch physicochemical properties of rice exposed to salinity stress at reproductive stage. J. Sci. Food Agric..

[13] Moradi F., Ismail A.M. (2007). Responses of photosynthesis, chlorophyll fluorescence and ROS-scavenging systems to salt stress during seedling and reproductive stages in rice. Anna. Bot..

[14] Singh R.K., Flowers T.J., Pessarakli M. (2010). The physiology and molecular biology of the effects of salinity on rice. Handbook of Plant and Crop Stress.

[15] Li Z.K., Xu J.L., Jenks M.A., Hasegawa P.M., Jain S.M. (2007). Breeding for drought and salt tolerant rice (*Oryza sativa* L.): Progress and perspectives. Advances in Molecular Breeding toward Drought and Salt Tolerance Crops.

[16] Guo R., Zhou J., Hao W., Gu F., Liu Q., Hao R.L., Xia X., Mao L. (2014). Germination, growth, chlorophyll fluorescence and ionic balance in linseed seedlings subjected to saline and alkaline stresses. Plant Prod. Sci..

[17] Yang C., Chong J., Li C., Kim C., Shi D., Wang D. (2007). Osmotic adjustment and ion balance traits of an alkali resistant halophyte *Kochia sieversiana* during adaptation to salt and alkali conditions. Plant Soil.

[18] Ishimaru Y., Kim S., Tsukamoto T., Oki H., Kobayashi T., Watanabe S., Matsuhashi S., Takahashi M., Nakanishi H., Mori S. (2007). Mutational reconstructed ferric chelate reductase confers enhanced tolerance in rice to iron deficiency in calcareous soil. Proc. Natl. Acad. Sci. USA.

[19] Marschner H. (1995). Mineral Nutrition of Higher Plants.

[20] Liu X., Zhang H., Jin Y., Wang M., Yang H., Ma H., Jiang C., Liang Z. (2019). Abscisic acid primes rice seedlings for enhanced tolerance to alkaline stress by upregulating antioxidant defense and stress tolerance-related genes. Plant Soil.

[21] Liu Y., Chen X., Xue S., Quan T., Cui D., Han L., Cong W., Li M., Yun D., Liu B. (2021). Set domain group 721 protein functions in saline-alkaline stress tolerance in the model rice variety Kitaake. Plant Biotechnol. J..

[22] Subudhi P.K., Shankar R., Jain M. (2020). Whole genome sequence analysis of rice genotypes with contrasting response to salinity stress. Sci. Rep..

[23] Kong W., Sun T., Zhang C., Deng X., Li Y. (2021). Comparative transcriptome analysis reveals the mechanisms underlying differences in salt tolerance between *indica* and *japonica* rice at seedling stage. Front. Plant Sci..

[24] Chapagain S., Concepcion J., Pruthi R., Singh L., Famoso A., Subudhi P.K. (2022). Genetic variation among the salinity tolerant breeding lines identified from two multi-parent advanced generation introgression line populations in rice (*Oryza sativa*). J. Agron. Crop Sci..

[25] Ren Z., Gao J., Li L., Cai X., Huang W., Chao D., Zhu M., Wang Z., Luan S., Lin H. (2005). A rice quantitative trait locus for salt tolerance encodes a sodium transporter. Nat. Genet..

[26] Huang X.Y., Chao D.Y., Gao J.P., Zhu M.Z., Shi M., Lin H.X. (2009). A previously unknown zinc finger protein, DST, regulates drought and salt tolerance in rice via stomatal aperture control. Genes Dev..

[27] Wang H., Wu Z., Chen Y., Yang C., Shi D. (2011). Effects of salt and alkali stresses on growth and ion balance in rice (*Oryza sativa* L.). Plant Soil Environ..

[28] Lin Y., Ma J., Wu N., Qi F., Peng Z., Nie D., Yao R., Qi X., Slaski J., Yang F. (2022). Transcriptome study of rice roots status under high alkaline stress at seedling stage. Agronomy.

[29] Chen W., Cui P., Sun H., Guo W., Yang C., Jin H., Fang B., Shi D. (2009). Comparative effects of salt and alkali stresses on organic acid accumulation and ionic balance of seabuckthorn (*Hippophae rhamnoides* L.). Ind. Crops Prod..

[30] Li N., Sun J., Wang J., Liu H., Zheng H., Yang L., Liang Y., Li X., Zou D. (2017). QTL analysis for alkaline tolerance of rice and verification of a major QTL. Plant Breed..

[31] Liu J., Shabala S., Shabala L., Zhou M., Meinke H., Venkataraman G., Chen Z., Zeng F., Zhao Q. (2019). Tissue-specific regulation of Na^+^ and K^+^ transporters explains genotypic differences in salinity stress tolerance in rice. Front. Plant Sci..

[32] Masuda H., Aung M.S., Kobayashi T., Hamada T., Nishizawa N.K. (2019). Enhancement of iron acquisition in rice by the mugineic acid synthase gene with ferric iron reductase gene and *OsIRO2* confers tolerance in submerged and nonsubmerged calcareous soils. Front. Plant Sci..

[33] Li X., Zheng H., Wu W., Liu H., Wang J., Jia Y., Li J., Yang L., Lei L., Zou D. (2020). QTL mapping and candidate gene analysis for alkali tolerance in *japonica* rice at the bud stage based on linkage mapping and genome-wide association study. Rice.

[34] Qi D., Guo G., Lee M., Zhang J., Cao G., Zhang S., Suh S., Zhou Q., Han L. (2008). Identification of quantitative trait loci for the dead leaf rate and the seedling dead rate under alkaline stress in rice. J. Genet. Genom..

[35] Liang J., Qu Y., Yang C., Ma X., Cao G., Zhao Z., Zhang S., Zhang T., Han L. (2015). Identification of QTLs associated with salt or alkaline tolerance at the seedling stage in rice under salt or alkaline stress. Euphytica.

[36] Singh L., Coronejo S., Pruthi R., Chapagain S., Subudhi P.K. (2022). Integration of QTL mapping and whole genome sequencing identifies candidate genes for alkalinity tolerance in rice (*Oryza sativa*). Int. J. Mol. Sci..

[37] Guan Q., Ma H., Wang Z.J., Wang Z.Y., Liu S.K. (2016). A rice LSD1-like-type ZFP gene *OsLOL5* enhances saline-alkaline tolerance in transgenic *Arabidopsis thaliana*, yeast and rice. BMC Genom..

[38] Moon H., Kim Y.A., Shin R., Park C.J. (2022). Nucleus-encoded thylakoid protein, OsY3IP1, confers enhanced tolerance to saline and alkaline stresses in rice. Rice Sci..

[39] Wang B., Xie G., Liu Z., He R., Han J., Huang S., Liu L., Cheng X. (2019). Mutagenesis reveals that the *OsPPa6* gene is required for enhancing the alkaline tolerance in rice. Front. Plant Sci..

[40] Li Q., Ma C., Tai H., Qiu H., Yang A. (2020). Comparative transcriptome analysis of two rice genotypes differing in their tolerance to saline-alkaline stress. PLoS ONE.

[41] Cheng H.T., Jiang H., Xue D.W., Guo L.B., Zeng D.L., Zhang G.H., Qian Q. (2008). Mapping of QTL underlying tolerance to alkali at germination and early seedling stages in rice. Acta Agron. Sin..

[42] Mei S., Zhang G., Jiang J., Lu J., Zhang F. (2022). Combining genome-wide association study and gene-based haplotype analysis to identify candidate genes for alkali tolerance at the germination stage in rice. Front. Plant Sci..

[43] Rieseberg L.H., Archer M.A., Wayne R.K. (1999). Transgressive segregation, adaptation and speciation. Heredity.

[44] Bhadru D., Rao V.T., Mohan Y.C., Bharathi D. (2012). Genetic variability and diversity studies in yield and its component traits in rice (*Oryza sativa* L.). SABRAO J. Breed. Genet..

[45] Amoah N.K.A., Akromah R., Kena A.W., Manneh B., Dieng I., Bimpong I.K. (2020). Mapping QTLs for tolerance to salt stress at the early seedling stage in rice (*Oryza sativa* L.) using a newly identified donor ‘Madina Koyo’. Euphytica.

[46] De Leon T.B., Linscombe S., Subudhi P.K. (2017). Identification and validation of QTLs for seedling salinity tolerance in introgression lines of a salt tolerant rice landrace ‘Pokkali’. PLoS ONE.

[47] Puram V.R.R., Ontoy J., Linscombe S., Subudhi P.K. (2017). Genetic dissection of seedling stage salinity tolerance in rice using introgression lines of a salt tolerant landrace Nona Bokra. J. Hered..

[48] Shabala S., Cuin T.A. (2008). Potassium transport and plant salt tolerance. Physiol. Plantar..

[49] Arif M.R., Islam M.T., Robin A.H.K. (2019). Salinity stress alters root morphology and root hair traits in *Brassica napus*. Plants.

[50] Xu N., Chu Y., Chen H., Li X., Wu Q., Jin L., Wang G., Huang J. (2018). Rice transcription factor OsMADS25 modulates root growth and confers salinity tolerance via the ABA-mediated regulatory pathway and ROS scavenging. PLoS Genet..

[51] De Leon T.B., Linscombe S., Subudhi P.K. (2016). Molecular dissection of seedling salinity tolerance in rice (*Oryza sativa* L.) using a high-density GBS-based SNP linkage map. Rice.

[52] De Leon T.B., Linscombe S., Gregorio G., Subudhi P.K. (2015). Genetic variation in Southern USA rice genotypes for seedling salinity tolerance. Front. Plant Sci..

[53] Puram V.R.R., Ontoy J., Subudhi P.K. (2018). Identification of QTLs for salt tolerance traits and prebreeding lines with enhanced salt tolerance in an introgression line population of rice. Plant Mol. Biol. Rep..

[54] Wang Z., Chen Z., Cheng J., Lai Y., Wang J., Bao Y., Huang J., Zhang H. (2012). QTL analysis of Na^+^ and K^+^ concentrations in roots and shoots under different levels of NaCl stress in rice (*Oryza sativa* L.). PLoS ONE.

[55] Wang Z., Cheng J., Chen Z., Huang J., Bao Y., Wang J., Zhang H. (2012). Identification of QTLs with main, epistatic and QTL × environment interaction effects for salt tolerance in rice seedlings under different salinity conditions. Theor. Appl. Genet..

[56] Lin W., Wang Y., Liu X., Shang J., Zhao L. (2021). *OsWAK112*, a wall-associated kinase, negatively regulates salt stress responses by inhibiting ethylene production. Front. Plant Sci..

[57] Eryong C., Bo S. (2022). OsABT, a rice WD40 domain-containing protein, is involved in abiotic stress tolerance. Rice Sci..

[58] Tran L.S.P., Mochida K. (2010). Identification and prediction of abiotic stress responsive transcription factors involved in abiotic stress signaling in soybean. Plant Signal. Behav..

[59] Li N., Liu H., Sun J., Zheng H., Wang J., Yang L., Zhao H., Zou D. (2018). Transcriptome analysis of two contrasting rice cultivars during alkaline stress. Sci. Rep..

[60] Solomon M., Belenghi B., Delledonne M., Menachem E., Levine A. (1999). The involvement of cysteine proteases and protease inhibitor genes in the regulation of programmed cell death in plants. Plant Cell.

[61] Park J.J., Yi J., Yoon J., Cho L.H., Ping J., Jeong H.J., Cho S.K., Kim W.T., An G. (2011). OsPUB15, an E3 ubiquitin ligase, functions to reduce cellular oxidative stress during seedling establishment. Plant J..

[62] Liu C.W., Hsu Y.K., Cheng Y.H., Yen H.C., Wu Y.P., Wang C.S., Lai C.C. (2012). Proteomic analysis of salt-responsive ubiquitin-related proteins in rice roots. Rapid Commun. Mass Spectrom..

[63] Berrin J.G., Pierrugues O., Brutesco C., Alonso B., Montillet J.L., Roby D., Kazmaier M. (2005). Stress induces the expression of *AtNADK-*1, a gene encoding a NAD(H) kinase in *Arabidopsis thaliana*. Mol. Genet. Genom..

[64] Cha-Um S., Supaibulwattana K., Kirdmanee C. (2009). Comparative effects of salt stress and extreme pH stress combined on glycine betaine accumulation, photosynthetic abilities and growth characters of two rice genotypes. Rice Sci..

[65] Karan R., De Leon T., Biradar H., Subudhi P.K. (2012). Salt stress induced variation in DNA methylation pattern and its influence on gene expression in contrasting rice genotypes. PLoS ONE.

[66] Finatto T., de Oliveira A.C., Chaparro C., da Maia L.C., Farias D.R., Woyann L.G., Mistura C.C., Soares-Bresolin A.P., Llauro C., Panaud O. (2015). Abiotic stress and genome dynamics: Specific genes and transposable elements response to iron excess in rice. Rice.

[67] Wang X., Li B.B., Ma T.T., Sun L.Y., Tai L., Hu C.H., Liu W.T., Li W.Q., Chen K.M. (2020). The NAD kinase *OsNADK1* affects the intracellular redox balance and enhances the tolerance of rice to drought. BMC Plant Biol..

[68] Sabouri H., Sabouri A. (2008). New evidence of QTLs attributed to salinity tolerance in rice. Afr. J. Biotechnol..

[69] Negi P., Rai A.N., Suprasanna P. (2016). Moving through the stressed genome: Emerging regulatory roles for transposons in plant stress response. Front. Plant Sci..

[70] Joly-Lopez Z., Forczek E., Vello E., Hoen D.R., Tomita A., Bureau T.E. (2017). Abiotic stress phenotypes are associated with conserved genes derived from transposable elements. Front. Plant Sci..

[71] Grandbastien M.A., Lucas H., Morel J.B., Mhiri C., Vernhettes S., Casacuberta J.M. (1997). The expression of the tobacco *Tnt1* retrotransposon is linked to plant defense responses. Genetica.

[72] Bui Q.T., Grandbastien M.A., Grandbastien M.A., Casacuberta J.M. (2012). LTR retrotransposons as controlling elements of genome response to stress?. Plant Transposable Elements: Impact on Genome Structure and Function Topics in Current Genetics.

[73] Linscombe S., Jodari F., Bollich P., Groth D., White L., Chu Q., Dunand R., Sanders D. (2000). Registration of “Cocodrie” rice. Crop Sci..

[74] Casartelli A., Riewe D., Hubberten H.M., Altmann T., Hoefgen R., Heuer S. (2018). Exploring traditional *aus*-type rice for metabolites conferring drought tolerance. Rice.

[75] Jones J.B., Case V.W., Westerman R.L. (1990). Sampling, handling, and analyzing plant tissue samples. Soil Testing and Plant Analysis.

[76] R Foundation (2005). R: A Language and Environment for Statistical Computing, Reference Index Version 2.2.1.

[77] SAS Institute Inc. (2012). SAS®9.4 System Options: Reference.

[78] Holland J.B., Nyquist W.E., Cervantes-Martínez C.T. (2003). Estimating and interpreting heritability for plant breeding: An update. Plant Breed. Rev..

[79] Murray M.G., Thompson W.F. (1980). Rapid isolation of high molecular weight plant DNA. Nucleic Acids Res..

[80] Elshire R.J., Glaubitz J.C., Poland J.A., Kawamoto K., Buckler E.S., Mitchell S.E. (2011). A robust, simple genotyping-by-sequencing (GBS) approach for high diversity species. PLoS ONE.

[81] Glaubitz J.C., Casstevens T.M., Lu F., Harriman J., Elshire R.J., Sun Q., Buckler E.S. (2014). TASSEL-GBS: A high capacity genotyping by sequencing analysis pipeline. PLoS ONE.

[82] Langmead B., Salzberg S. (2012). Fast gapped-read alignment with Bowtie 2. Nat. Methods.

[83] Meng L., Li H., Zhang L., Wang J. (2015). QTL IciMapping: Integrated software for genetic linkage map construction and quantitative trait locus mapping in biparental populations. Crop J..

[84] Kosambi D.D. (1944). The estimation of map distances from recombination values. Ann. Eugen..

[85] Li H., Durbin R. (2009). Fast and accurate short read alignment with Burrows-Wheeler transform. Bioinformatics.

[86] Patel R.K., Jain M. (2012). NGS QC Toolkit: A toolkit for quality control of next generation sequencing data. PLoS ONE.

[87] Danecek P., Bonfield J.K., Liddle J., Marshall J., Ohan V., Pollard M.O., Whitwham A., Keane T., McCarthy S.A., Davies R.M. (2021). Twelve years of SAMtools and BCFtools. Gigascience.

[88] Van der Auwera G.A., O’Connor B.D. (2020). Genomics in the Cloud: Using Docker, GATK, and WDL in Terra.

[89] Poplin R., Ruano-Rubio V., DePristo M.A., Fennel T.J., Carneiro M.O., Van der Auwera G.A., Kling D.E., Gauthier L.D., Levy-Moonshine A., Roazen D. (2018). Scaling accurate genetic variant discovery of tens of thousands of samples. BioRxiv.

[90] Cingolani P., Platts A., Wang L.L., Coon M., Nguyen T., Wang L., Land S.J., Lu X., Ruden D.M. (2012). A program for annotating and predicting the effects of single nucleotide polymorphisms, SnpEff: SNPs in the genome of Drosophila melanogaster strain w^1118^; iso-2; iso-3. Fly.

[91] Subudhi P.K., Garcia R.S., Coronejo S., Tapia R. (2020). Comparative transcriptional profiling of root tissues in two rice genotypes reveals differential expressed genes associated with root architecture under nitrogen stress. Int. J. Mol. Sci..

[92] Livak K., Schmittgen T. (2001). Analysis of relative gene expression data using real-time quantitative PCR and the 2^−∆∆CT^ method. Methods.

