# Supplementary material for: Genetic Dissection of Alkalinity Tolerance at the Seedling Stage in Rice (Oryza sativa) Using a High-Resolution Linkage Map
Source: Plants (Basel). 2022 Dec 2;11(23):3347. doi: 10.3390/plants11233347 (PMC9738157; doi:10.3390/plants11233347)
Supplement: Supplementary file 1 [file plants-11-03347-s001.zip › Supplementary Figure S1.pdf]

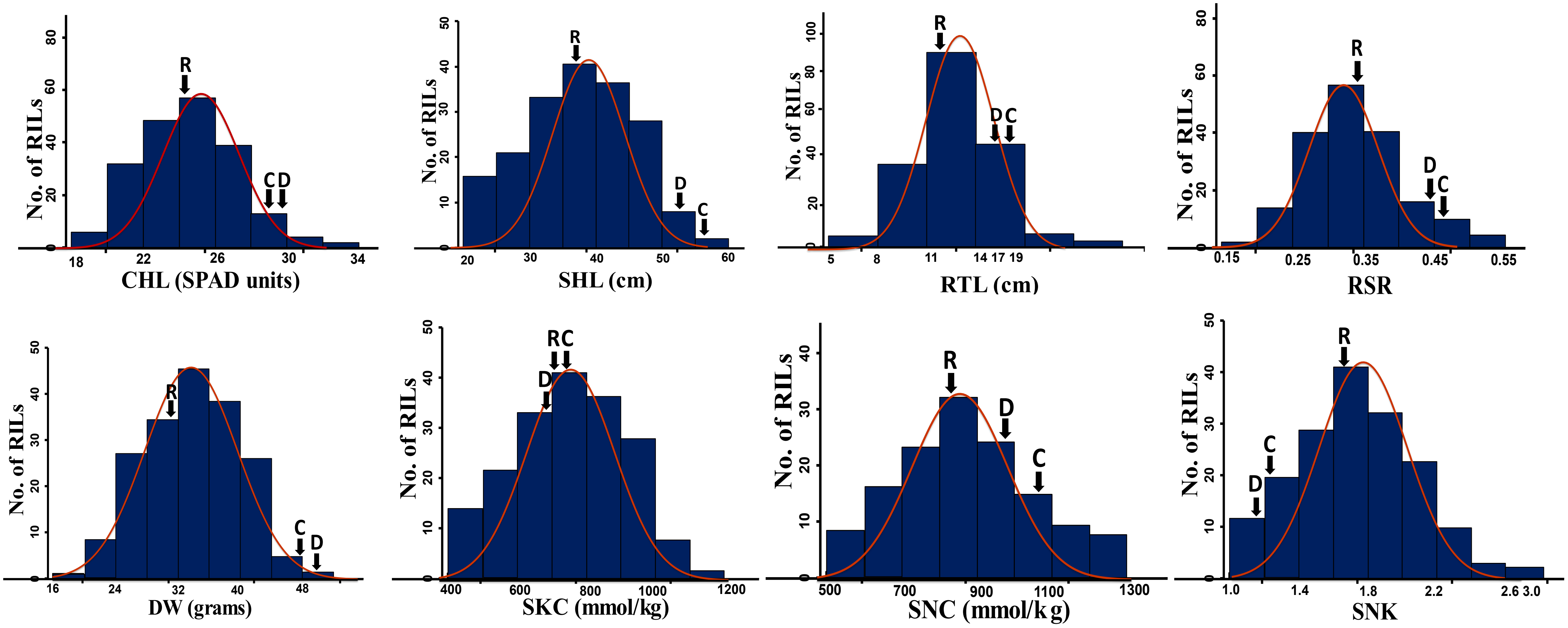

Figure S1. Frequency distribution of various morphological and physiological traits of Cocodrie x Dular RILs under a control condition at the seedling stage with arrowheads indicating the trait means of Cocodrie (C), Dular (D), and RIL population (R). CHL – chlorophyll content; SHL – shoot length; RTL – root length; RSR – root to shoot ratio; DW – shoot dry weight; SKC – shoot K^+ concentration; SNC – shoot Na^+ concentration; SNK – shoot Na^+:K^+ ratio.
