# Supplementary material for: Genetic Dissection of Alkalinity Tolerance at the Seedling Stage in Rice (Oryza sativa) Using a High-Resolution Linkage Map
Source: Plants (Basel). 2022 Dec 2;11(23):3347. doi: 10.3390/plants11233347 (PMC9738157; doi:10.3390/plants11233347)
Supplement: Supplementary file 1 [file plants-11-03347-s001.zip › Supplementary Figure S2.pdf]

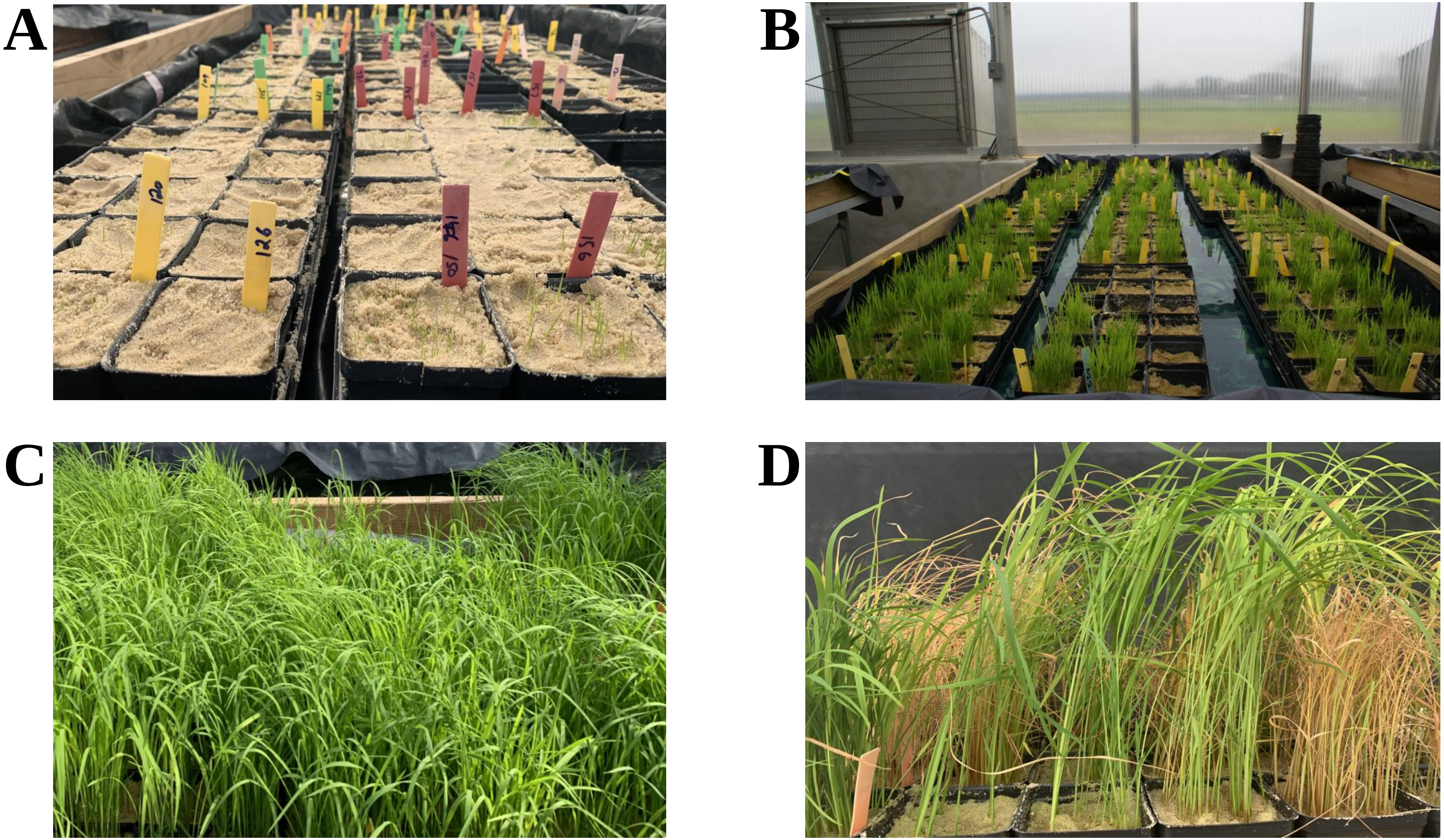

Figure S2. Alkalinity tolerance screening of RIL mapping population and parents in sand culture in the greenhouse experiment. A - Experimental setup for alkalinity stress screening at the seedling stage; B - one week old seedlings after germination; C - Performance of RILs under control experiment; D - Performance of RILs after 2 weeks of exposure to alkalinity stress.
