# Supplementary material for: Genetic Dissection of Alkalinity Tolerance at the Seedling Stage in Rice (Oryza sativa) Using a High-Resolution Linkage Map
Source: Plants (Basel). 2022 Dec 2;11(23):3347. doi: 10.3390/plants11233347 (PMC9738157; doi:10.3390/plants11233347)
Supplement: Supplementary file 1 [file plants-11-03347-s001.zip › Table S1.pdf]

Table S1. Mean values for various morpho-physiological traits at the seedling stage under control environment in Cocodrie x Dular RIL population

Traits[a]	Cocodrie Mean	Dular Mean[b]	RIL Mean	Heritability
SNC (mmolkg^{-})	1225.38	1039.29^{ns}	1301.56	0.84
SKC (mmolkg^{-})	738.27	673.72^{ns}	692.53	0.93
SNK (ratio)	1.26	0.98^{ns}	1.17	0.68
AKT	1	1^{ns}	1.7	0.94
log_CHL	1.57	1.53^{ns}	1.56	0.87
log_SHL	1.55	1.53^{ns}	1.51	0.83
RTL (cm)	16.9	16.2^{ns}	17.63	0.89
DW (g)	48.3	50.1^{ns}	52.87	0.92
RSR (ratio)	0.51	0.48^{ns}	0.47	0.81

a

[a]SNC, shoot Na^+ concentration; SKC, shoot K^+ concentration; SNK, shoot Na^+:K^+ ratio; AKT, alkalinity tolerance score; log_CHL, log chlorophyll content; log_SHL, log shoot length; RTL, root length; DW, Dry weight; RSR, root to shoot ratio

[b]t-test between Cocodrie and Dular; ^{ns} Nonsignificant
