# Supplementary material for: Genetic Dissection of Alkalinity Tolerance at the Seedling Stage in Rice (Oryza sativa) Using a High-Resolution Linkage Map
Source: Plants (Basel). 2022 Dec 2;11(23):3347. doi: 10.3390/plants11233347 (PMC9738157; doi:10.3390/plants11233347)
Supplement: Supplementary file 1 [file plants-11-03347-s001.zip › Table S2.pdf]

Table S2. Additive QTLs for physiological and morphological traits under control environment at seedling stage identified by ICIM method in Cocodrie x Dular RIL population

Trait[a]	QTL[b]	Chr	Left_Marker	Right_Marker	LOD[c]	PVE (%)[d]	Additive effect	Increasing allele effect
CHL	*qCHL3.35*	3	S3_35332218	S3_35570746	2.2188	2.8087	-27.555	Dular
	qCHL7.01	7	S7_1101600	S7_1144731	20.3266	36.4626	10.6729	Cocodrie
SHL	*qSHL1.03*	1	S1_3665789	S1_3708821	21.7107	38.0288	10.5115	Cocodrie
	qSHL2.25	2	S2_25044454	S2_25045214	2.2189	2.809	-38.0441	Dular
	qSHL4.31	4	S4_31716088	S4_31775328	5.3272	7.4501	2.2043	Cocodrie
	qSHL5.25	5	S5_25515196	S5_25687880	5.3273	7.4504	44.0864	Cocodrie
	qSHL9.18	9	S9_18208683	S9_18332939	7.8913	16.9238	-0.0346	Dular
	qSHL12.16	12	S12_16498758	S12_16498772	5.3271	7.4503	55.1081	Cocodrie
RTL	*qRTL10.10*	10	S10_10182742	S10_10329302	3.0981	7.9091	0.4725	Cocodrie
	qRTL12.05	12	S12_5751404	S12_6224392	2.9213	8.1571	0.4751	Cocodrie
RSR	*qRSR1.28*	1	S1_28526959	S1_28526967	2.6652	3.7663	31.4875	Cocodrie
	qRSR6.13	6	S6_13805365	S6_14333027	21.7108	38.0285	5.0256	Cocodrie
SNC	*qSNC1.34*	1	S1_34605734	S1_34643510	2.0684	2.9119	34.3716	Dular
	qSNC3.10	3	S3_10549867	S3_10676606	2.0684	2.9111	27.4933	Dular
	qSNC9.22	9	S9_22504186	S9_22504216	2.7844	5.3441	0.0193	Dular
	qSNC12.01	12	S12_1055785	S12_1055788	2.6651	3.7664	39.3592	Dular
	qSNC12.24	12	S12_24823703	S12_24823709	2.6652	3.7659	1.5744	Dular
SKC	*qSKC2.30*	2	S2_30952914	S2_30823340	2.2187	2.8089	-1.9022	Cocodrie
	qSKC2.03	2	S2_3263428	S2_4408126	21.7108	21.0284	-125.6393	Cocodrie
	qSKC4.33	4	S4_33881790	S4_33875971	2.6652	3.7663	-31.4875	Cocodrie
	qSKC5.02	5	S5_2831482	S5_3374773	2.0236	4.7419	-0.3623	Cocodrie
	qSKC10.004	10	S10_460111	S10_584084	2.0684	2.9123	-1.3749	Cocodrie
SNK	*qSNK1.14*	1	S1_14938489	S1_15440503	5.7533	8.4379	-52.2813	Cocodrie
	qSNK7.05	7	S7_5062144	S7_5182685	15.8854	12.1423	11.7262	Dular
	qSNK9.19	9	S9_19167572	S9_19175157	2.1118	3.082	31.7373	Dular
	qSNK9.11	9	S9_11236368	S9_11348590	2.0627	2.7287	-41.7847	Cocodrie

[a]CHL, chlorophyll content; SHL, shoot length; RTL, root length; RSR, root to shoot ratio; SNC, shoot Na^+ concentration; SKC, shoot K^+ concentration.; SNK, shoot Na^+:K^+ ratio

[b]*qCHL*, *qSHL*, *qRTL*, *qRSR*, *qSNC*, *qSKC*, *qSNK* correspond to QTLs for chlorophyll content, shoot length, root length, root to shoot ratio, shoot Na^+ concentration, shoot K^+ concentration, and shoot Na^+:K^+ ratio, respectively. The number before the decimal represent the chromosome number and the number after the decimal indicate the physical position of the QTLs in mega base pair.

[c]LOD, logarithm of odds

[d]PVE (%), percentage phenotypic variance explained by the QTL
